# Reliable estimation of tree branch lengths using deep neural networks

**DOI:** 10.1371/journal.pcbi.1012337

**Published:** 2024-08-05

**Authors:** Anton Suvorov, Daniel R. Schrider

**Affiliations:** 1 Department of Biological Sciences, Virginia Tech, Blacksburg, Virginia, United States of America; 2 Department of Genetics, University of North Carolina at Chapel Hill, Chapel Hill, North Carolina, United States of America; Ecole Normale Superieure, FRANCE

## Abstract

A phylogenetic tree represents hypothesized evolutionary history for a set of taxa. Besides the branching patterns (i.e., tree topology), phylogenies contain information about the evolutionary distances (i.e. branch lengths) between all taxa in the tree, which include extant taxa (external nodes) and their last common ancestors (internal nodes). During phylogenetic tree inference, the branch lengths are typically co-estimated along with other phylogenetic parameters during tree topology space exploration. There are well-known regions of the branch length parameter space where accurate estimation of phylogenetic trees is especially difficult. Several novel studies have recently demonstrated that machine learning approaches have the potential to help solve phylogenetic problems with greater accuracy and computational efficiency. In this study, as a proof of concept, we sought to explore the possibility of machine learning models to predict branch lengths. To that end, we designed several deep learning frameworks to estimate branch lengths on fixed tree topologies from multiple sequence alignments or its representations. Our results show that deep learning methods can exhibit superior performance in some difficult regions of branch length parameter space. For example, in contrast to maximum likelihood inference, which is typically used for estimating branch lengths, deep learning methods are more efficient and accurate. In general, we find that our neural networks achieve similar accuracy to a Bayesian approach and are the best-performing methods when inferring long branches that are associated with distantly related taxa. Together, our findings represent a next step toward accurate, fast, and reliable phylogenetic inference with machine learning approaches.

## Author summary

Phylogenetic trees that delineate organismal relationships serve as a cornerstone structure for almost any basic research leveraging evolutionary information. Besides the tree topology, phylogeneticists are concerned with estimating other fundamental phylogenetic parameters such as the lengths of each branch in the tree. The tree branch lengths are proportional to evolutionary distances between taxa, with long branches representing distantly related taxa and/or accelerated evolution, whereas short branches are indicative of close taxonomic relationships and/or slower evolutionary rates. There is a plethora of phylogenetic methods that can infer branch lengths from sequence data, but they typically exhibit elevated error rates within certain regions of the branch length parameter space and thus in some cases may provide poor estimates. Here, as a proof-of-concept study, we explored the possibility of using artificial neural networks (ANNs) to accurately estimate branch lengths directly from sequence data or its summaries. We show that ANNs can reliably infer branch lengths with accuracy on par with or even better than traditional methods such as Bayesian and maximum likelihood approaches, especially when branches are long. We argue that further investigation of machine learning methods could lead to marked improvements in phylogenetic inference.

## Introduction

Phylogenetic inference is primarily concerned with the estimation of different evolutionary parameters from multiple sequence alignments (MSA) of molecular data using a wide range of statistical approaches. One of the major objectives is to estimate a tree topology, which can be viewed as an unconventional parameter [1] that determines historical relationships of the taxa under consideration. Other fundamental parameters of interest are the branch lengths that represent evolutionary (genetic) distances between the nodes of this tree. Typically, the branch lengths, ***b***, of a tree represent relative distances expressed in terms of the time duration *t* and the substitution rate *θ*. The parameters *t* and *θ* are not identifiable individually [2,3] unless there is additional information available, such as fossil record, dated geological events and/or direct observation of mutation rates [4]. Despite the wealth of research literature in phylogenetics that focuses on tree estimation methods, the problem of branch length inference traditionally receives less attention than topology inference. To estimate ***b*** under maximum-likelihood (ML) criteria, these branch lengths are simultaneously optimized on a fixed tree topology to achieve an optimal likelihood score [5]. Typically, the length estimates of different branches in a tree are considered to be independent quantities, allowing substitution to be modeled as a Markov process as is done under ML criteria [6].

In recent years machine learning methods, and deep learning frameworks in particular, have gained an increasing amount of attention from the phylogenetic community. A wide range of phylogenetic tasks were tackled from the machine learning perspective including tree topology estimation [7,8], the investigation of zones of phylogenetic bias [7,9], the identification of autocorrelation in evolutionary rates [40], model selection [10], taxon placement on existing trees [50] and improving tree topology search [11].

Estimation of ***b*** is a critical step in phylogenetic inference because a great deal of statistical methods of divergence time estimation critically depend on the accuracy of these estimates [12] as absolute geological time *t* is confounded in ***b***. If ***b*** is not estimated accurately, then subsequent calibration analyses will in turn be incorrect and may result in erroneous inferences from a wide range of evolutionary models. These may include phylodynamic analyses of viral outbreaks where branch lengths provide critical information about lineage turnover [13] or estimation of macroevolutionary parameters such as speciation and extinction rates from time-calibrated phylogenies [14]. Thus, inference of ***b*** will exert a “domino effect”, whether positive of negative, on the numerous downstream evolutionary analyses.

Here, we provide a machine learning framework to estimate ***b*** ether directly from an MSA using convolutional neural networks, or from summaries of the MSA, such as site pattern frequencies, using a multilayer perceptron. We further compare the performance of these methods with the most commonly used methods of branch length inference—the maximum likelihood and Bayesian approaches. Our simulation study shows that machine learning methods provide reliable estimates of ***b*** often outperforming these traditional methods and excelling in certain difficult regions of the branch-length parameter space.

## Methods

Our simulation procedures were divided into two consecutive tasks: (i) the simulation of tree branch lengths (***b***) and (ii) the generation of corresponding MSAs. Then, simulated ***b*** and MSAs were used to compare the performance of branch-length inference by maximum likelihood to our deep learning approaches. We examined a variety of different combinations of parametric distributions for ***b*** and models of the substitution process. We describe these in detail below.

### Uniform and exponential models

We simulated branch lengths for unrooted quartet topologies under several uniform distributions with different minimum and maximum parameter values: *U*(0,0.001), *U*(0.001,0.01), *U*(0.01,0.1), *U*(0.1,1) and *U*(1,10). We also simulated ***b*** under an exponential distribution, which is viewed as a “biologically interpretable” probability model [15]. In particular, we examined three exponential distributions with mean (*α*) of 1, 0.1 and 0.01, respectively. Each branch length in a simulated tree was drawn independently from the same parametric distribution. Additionally, we used *U*(0.1,1) and *U*(1,2) distributions to simulate data for the “misspecified branch length distribution” experiments, whereas *Exp*(0.1) distribution was used to generate ***b*** for 8-taxon trees with different degrees of topology balance (**Table 1**).

**Table 1:** Summary of the multiple sequence alignment simulation experiments that were performed in this study using different branch length distributions, substitution models and tree topologies. *U*(*min*, *max*) = uniform distribution with the corresponding minimum and maximum parameter values. *Exp*(*α*) = exponential distribution with the corresponding mean parameter *α*. *ϵ* is the relative extinction parameter for the birth-death model (i.e. *μ*/*λ* where *μ* and *λ* are the death and birth parameters, respectively) and *a* is the age of the root node in Mya of the birth-death model. BL-space = branch-length heterogeneity space. Each simulation consists of a combination of a specific branch length distribution, substitution model and tree topology. For example, the “exponential” experiment includes a total of six parameter combinations: all pairs of the three branch length distributions and two substitution models in the “Exponential” entry in the table.

Experiment	Branch length distribution parameters	Substitution models	Topology	Remarks
**Uniform**	U(0,0.001),U(0.001,0.01),U(0.01,0.1),U(0.1,1),U(1,10)	JC	unrooted 4 taxon (A,B,(C,D))	
**Exponential**	*Exp*(1), *Exp*(0,1), *Exp*(0.01)	JC, GTR	unrooted 4 taxon (A,B,(C,D))	
**BL-space**	Beta mixture	JC, GTR	unrooted 4 taxon (A,B,(C,D))	
**Birth-death**	*ϵ* = 0,0.5 or 0.9 *a* = 10,50,100 or 200 Clock rate = 0.001	3.3b, UNREST	rooted 4 taxon ((A,B),(C,D))	
**Substitution model misspecification**	*Exp*(1), *Exp*(0,1), *Exp*(0.01)	JC, GTR	unrooted 4 taxon (A,B,(C,D))	Train dataset was simulated under JC and applied to test dataset simulated under GTR and vice versa.
**Misspecified branch length distribution**	*U*(0.1,1), *U*(1,2)	JC	unrooted 4 taxon (A,B,(C,D))	Train dataset was simulated using *U*(0.1,1) and test dataset was simulated using *U*(1,2) and vice versa.
**Effects of tree topology balance**	*Exp*(0.1)	GTR	unrooted 8 taxon (A,B,((C,D),((E,F),(G,H)))) (A,B,((C,D),(E,(F,(G,H))))) (A,B,(C,(D,((E,F),(G,H))))) (A,B,(C,(D,(E,(F,(G,H))))))	

### Sampling from branch-length heterogeneity space

Additionally, to assess the impact of branch-length asymmetry on estimation accuracy, we simulated ***b*** from our previously described “branch-length heterogeneity space” [7] or “BL-space”. Briefly, to generate these data we simulated 10^6^ quartet topologies with randomly assigned branch lengths drawn from the following mixture of beta distributions:

$$b∼w_{1}Beta(\alpha =\beta =0.1)+w_{2}Beta(\alpha =\beta =0.5)+w_{3}Beta(\alpha =\beta =1)$$

$$w_{1}=w_{2}=w_{3}=1/3$$

Then, we projected each tree branch length configuration onto a 2D space using three simple statistics: the sum of pairwise differences (PD) of branch lengths, total tree length (L) and sum of lengths of neighboring branches (NS) as detailed in Suvorov et al. (2020) [7]. Finally, we uniformly sampled branch length configurations from this 2D space. This approach allowed us to simulate trees with various degree of branch length heterogeneity, including trees from regions of the parameter space that are known to cause biased estimation of tree topologies, such as Felsenstein [16] and Farris [17] zones.

### Birth-death model

We simulated trees under the birth-death (BD) process using a two-step procedure: 1) the generation of the absolute branch lengths ***b***_*a*_ using RevBayes v1.0.12 [18], followed by 2) the conversion of ***b***_*a*_ to relative branch lengths ***b*** using a strict clock model in NELSI v0.21 [19]. First, we generated ***b***_*a*_ from the following model:

$$f(b_{a}|n,T,\lambda ,\mu ,a\prime )$$

where *T* is a fixed tree topology, *n* is the number of taxa, *λ* and *μ* are the birth and death rates, respectively, and *a*′ is a root node age drawn from *U*(*a*, *a*+0.001). This tight uniform prior was used because we desired a fixed root node age, but RevBayes requires a prior distribution. In order to constrain *T* in RevBayes, we disallowed all tree topology move proposals, leaving only the scale and slide operators that change branch lengths only. We did not specify *λ* and *μ* directly, but instead specified the relative extinction parameter, i.e. *ϵ* = *μ*/*λ*, and then computed *λ* (Eq 1) and *μ* (Eq 2) using the method-of-moments estimator [20] for the net diversification rate, *r* (Eq 3), as follows:

$$\lambda =\frac{\hat{r}}{1-\epsilon }$$ (1)

$$\mu =\lambda -\hat{r}$$ (2)

$$\hat{r}=\lambda -\mu =\frac{log[\frac{n(1-\epsilon^{2})}{2}+2\epsilon +\frac{(1-\epsilon )\sqrt{n(n\epsilon^{2}-8\epsilon +2n\epsilon +n)}}{2}]-\log\left(2\right)}{a}$$ (3)

We used all pairwise combinations of the following BD parameter values: *ϵ* of 0, 05 and 0.9, and *a* of 10, 50, 100 and 200. Then, we performed Markov Chain Monte Carlo (MCMC) sampling in RevBayes to generate ***b***_*a*_ after discarding the first 5,000 generations as burn-in. Finally, ***b***_*a*_ were scaled to ***b*** in NELSI using strict clock model with a constant rate of 0.01 substitutions per site per million years, and no noise.

### Multiple sequence alignment simulations

MSAs were simulated using phylogenetic simulator AliSim [21] which is integrated into the IQ-TREE v2.2.0 software [22]. We used a wide range of DNA substitution models including time-reversible Markov models such as JC and GTR as well as non-reversible Lie Markov models [23], namely 3.3b and UNREST [24] (which is equivalent to 12.12 model in IQ-TREE). The free substitution rate parameters for GTR were drawn from *U*(0, 1), whereas for the UNREST model these parameters were drawn from *U*(0, 0.979) as they cannot exceed value of 0.98 for Lie Markov models (if this condition is not satisfied AliSim raises an error). The equilibrium base frequencies for JC and 3.3b were specified with 0 degrees of freedom, i.e. *π*_*A*_ = *π*_*G*_ = *π*_*C*_ = *π*_*T*_ = 1/4, whereas for GTR and UNREST base frequencies were unconstrained with 3 degrees of freedom and were drawn from *U*(0, 1) and then normalized in order to satisfy *π*_*A*_+*π*_*G*_+*π*_*C*_+*π*_*T*_ = 1. The among-site rate variation (+Γ) was used with every substitution model and was modeled using discrete Gamma model with four rate categories with the shape parameter drawn from *U*(0, 1). Thus, every MSA was simulated along a given tree (with branch lengths drawn as described above) using the specified substitution model parameters. Our complete set of simulation experiments and their respective combinations of branch length distributions, substitution models and tree topologies are summarized in **Table 1**.

### Deep learning architectures and training

For each simulation experiment shown in **Table 1**, we generated a training set consisting of 15 × 10^4^ trees with branch lengths drawn from a specified distribution, with the MSA for each tree then generated using AliSim. The length of each MSA was set to 1,000 sites. We treated each MSA as a matrix with rows corresponding to sequences and columns corresponding to sites in the alignment, and with each character in the alignment encoded as a number (“A”:0, “T”:1, “C”:2, “G”:3). Even though other encodings are possible, e.g. one-hot encoding [25], we adopted the current encoding scheme as it was successfully used in our previous study [7].

The main objective of a supervised machine learning algorithm is to learn mapping of inputs, ***X***, to outputs, ***Y***. In our case the MSAs, or a representation thereof, served as inputs, and the branch lengths ***b*** were our outputs. Since the goal is to predict positive real-valued ***b***∈ℝ^+^ from a given MSA, our task is a regression problem. Within this study we explored and compared behavior of two types of artificial neural networks trained to complete this task: a multilayer perceptron (MLP) and a convolutional neural network (CNN). We used the Keras v2.9.0 Python API (https://keras.io/) with TensorFlow v2.9.1 as the backend [26] to build, train and test these machine learning models.

As our input for the MLP, we constructed feature vectors ***X*** by counting site patterns found within an MSA and then divided these counts by the alignment length. There exist exactly 4^4^ = 256 such possible patterns (i.e., features) for an alignment of four taxa, as gap characters were not examined in this study. For the “effects of tree topology balance” experiments we extracted site patterns directly from 8-taxon alignments resulting in 4^8^ = 65,536 features. Our MLP architecture comprised an input layer, three fully connected dense hidden layers of 1,000 neurons each with rectified linear unit (ReLU) activation, and an output layer with the number of neurons set to the number of tree branches and with linear activation. Additionally, we used the dropout regularization technique with a rate of 0.15 applied to every hidden layer.

The CNN architectures built for this study used network architectures/hyperparameters similar to the ones utilized in [7] with some modifications. The CNN uses entire MSAs as input ***X*** and extracts abstract features by performing several convolutional and pooling operations. Our CNN contained an input layer, six convolutional layers consisting of 1024, 1024, 1024, 128, 128 and 128 filters, respectively. Each convolutional layer used ReLU activation and was followed by a pooling layer. We set the kernel size for the first convolution operation to 4 × 1, reasoning that it would potentially capture a phylogenetic signal by striding across the MSA and examining one column of the alignment at a time, and the subsequent convolution steps had each kernel sizes of 1 × 2. The first average-pooling operation size was set to 1 × 1 (i.e., no pooling), with subsequent pooling steps having sizes of 1 × 4, 1 × 4, 1 × 4, 1 × 2 and 1 × 2. Finally, the output of the last pooling layer was flattened and passed to a fully connected dense layer of 1,000 neurons with ReLU activation, which in turn was connected to the output layer, whose number of neurons was equal to the number of tree branches. Again, the output layer used linear activation. Here, we did not use batch normalization or dropout regularization, as we observed that these tended to negatively affect learning stability and the accuracy of predicted branch lengths.

Machine learning regression models can suffer from systematic biases [27,28] caused by overestimation and underestimation of small and large output variables ***Y***, respectively, and/or by a transformation of original output variables ***Y***. We observed the latter form of bias in our initial experiments, and therefore sought to correct this using the “regression of observed on estimated values” (ROE) technique [27]. This approach includes two steps: (i) a *trained* neural network model is used to predict output values $\hat{Y}$ on training dataset ***X***, and (ii) these estimated values $\hat{Y}$ from the training set are used in turn to train a separate regression model seeking to better estimate the true output values ***Y***. To build and train this second regression model, we used a multiple linear regression model implemented in Keras (i.e. an input layer, whose size is equal to the number of tree branches, connected to an output layer of the same size and which uses linear activation).

When training our MLP, CNN, ROE regression models, 10% of the training dataset was set aside for validation. We used mean squared error (MSE) as our loss function. We initially found that our neural networks occasionally predicted negative branch lengths whose values were less than zero. We therefore performed square root transformation of ***b*** prior to training, and then squared the predicted ***b*** after running the network, thereby ensuring that predicted ***b***∈ℝ^+^. The networks’ parameters (weights) were updated during training using the adaptive moment estimation (Adam) optimizer with batches sizes of 100, 32, and 32 for the MLP, CNN and ROE architectures, respectively. To prevent a neural network from overfitting, we used early stopping by monitoring validation loss metric during training and used a patience value of 10, causing training to terminate whenever loss on the validation set failed to improve for 10 consecutive training epochs. Loss was considered to have improved if it decreased by at least 0.0001 relative to the minimum loss observed across all previous epochs in the run (i.e. min_delta = 0.0001).

**Fig 1:**
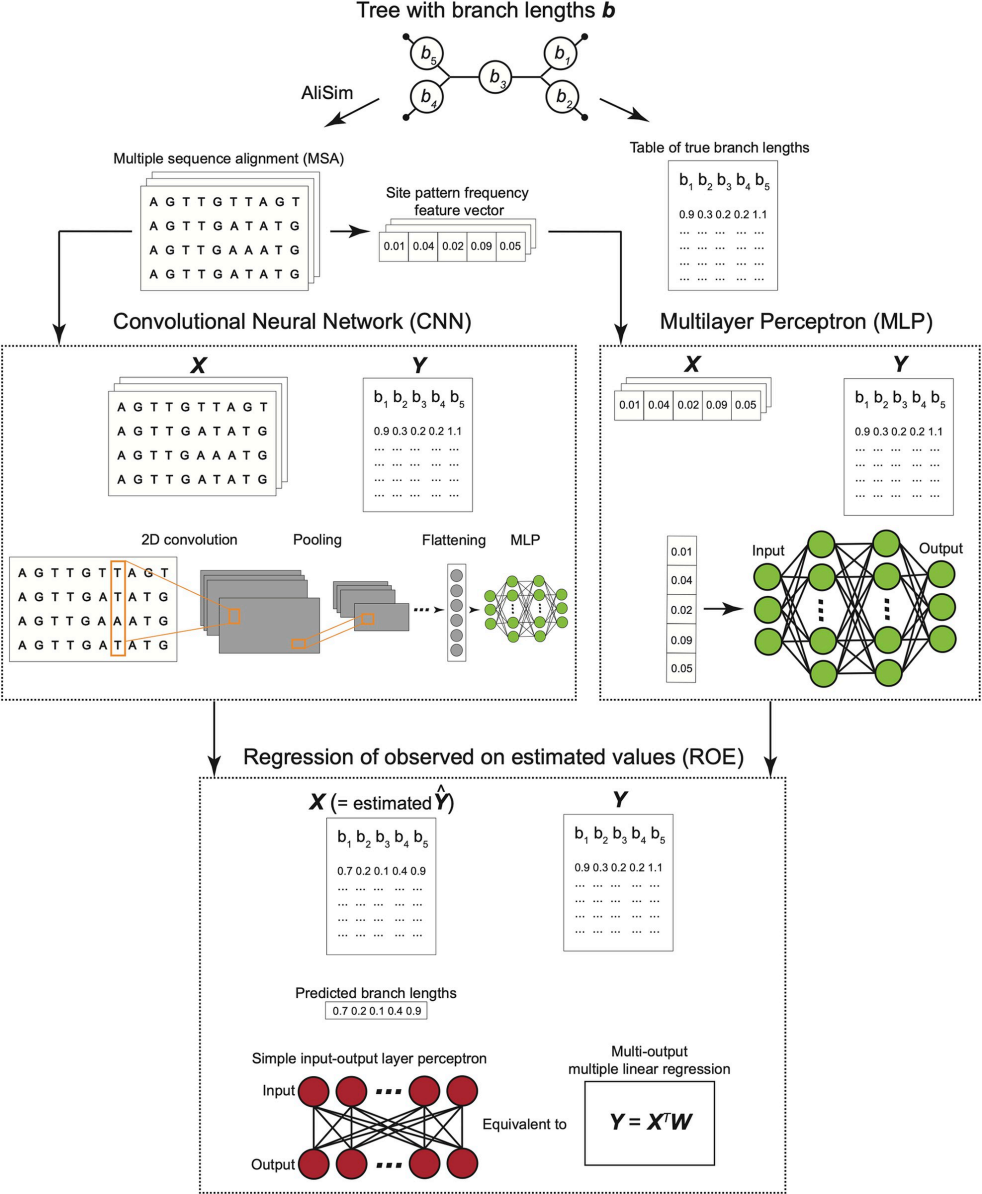
Flowchart summary of simulation and artificial neural network training procedures. After generating trees with branch lengths, multiple sequence alignments (MSA) were simulated with AliSim. From each MSA site pattern frequencies were calculated. MSAs (or site frequency vectors) together with the true branch lengths were used as input **X** and target **Y** in the convolutional neural network (CNN) and multilayer perceptron (MLP) architectures. The outputs estimated by the CNN (or MLP) on the training dataset were used as an input **X** in the additional “Regression of observed on estimated values” step, where the true values were again used as target **Y**. Once all networks were trained, they were subsequently used to predict branch lengths from a testing dataset.

In total, we constructed four deep learning models: MLP, CNN, MLP-ROE, and CNN-ROE where the latter two were trained by adding the linear regression step onto the previously trained MLP and CNN regression models. The flowchart in **Fig 1** summarizes the steps of the training procedure and the nature of our input and target variables.

### Testing procedures

We compared the performance of the deep learning architectures described above to a standard method of branch length tree estimation: maximum likelihood (ML) estimation as implemented in IQ-TREE. To that end, for each simulation experiment we generated an additional test set of 10,000 trees, each with branch lengths ***b***, and their corresponding MSA’s. In order to estimate branch lengths on a fixed tree topology in IQ-TREE, we generally used the same substitution model that had been specified to generate the input MSA. In the “Model misspecification” experiments (see **Table 1**), however, we used a different substitution model in IQ-TREE, and for training our networks, than that of the test data, allowing us to observe the impact of model misspecification on branch-length estimation accuracy. Again, we also used branch length distributions that differ between the training and test data for the “Misspecified branch length distribution” experiments, although we note that this has no impact on maximum likelihood estimation, which does not require a pre-specified distribution of branch lengths.

Additionally, we performed Bayesian branch length estimation using MrBayes 3.2.7a [29] for the “exponential” experiments under the JC model (**Table 1**). All Bayesian estimates were run on a fixed tree topology specifying the JC+Γ model with exponential priors for branch lengths identical to the exponential distributions used in simulations, e.g. if ***b*** were drawn from *Exp*(1), then prior for ***b*** in a Bayes run was also set to *Exp*(1). For each Bayes analysis we used three independent MCMC chains of 10^5^ generations, sampling every 100^th^ generation and the burn-in period of 1000 generations. For our length point estimate for a specific branch, we used the median of a posterior distribution.

We summarized the performance of each method by measuring the mean squared error (MSE) and mean absolute error (MAE) for each estimated branch length and total tree length (i.e., the sum of the branch length vector ***b***). MSE is a useful criterion to compare efficiency of estimators, i.e. our branch length inference methods, where lower MSE values indicate higher efficiency. Additionally, we quantified bias by comparing the empirical cumulative distribution functions (eCDF) of observed and estimated ***b***. First, error (*ε*) was calculated using the sample quantile function *Q* for 100 quantiles of the true branch lengths (Eq 4):

$$\varepsilon_{i}=Q_{i}(b)-Q_{i}(\hat{b})$$ (4)

Where ***b*** and b^ represent the true and estimated branch lengths, respectively, and *i* denotes the *i*^th^ quantile. Then, bias (Eq 5) was computed as:

$$bias=\sqrt{\frac{1}{n}\sum_{i}{\varepsilon_{i}}^{2}}$$ (5)

where *n* is equal to the total number of quantiles (i.e. 100). Further, the distributions of observed and estimated ***b*** were statistically compared using a two-sample Kolmogorov-Smirnov (KS) test. We report KS test statistic *D*, which together with *bias* measure (largest) vertical and (average) horizontal distances, respectively, between true and observed eCDF’s. The relationships between observed and predicted values of ***b*** were also evaluated using the Spearman rank correlation test (SR) and its estimated correlation coefficient *ρ*. We also examined whether our methods tend to overestimate or underestimate ***b*** within certain parameter spaces. Specifically, we compared distributions of residuals, i.e. differences between true and estimated ***b***, by calculating the ratio of residuals with “–” and “+” signs, where the ratios of >1would suggest overestimation, whereas the ratios of <1 are indicative of underestimation. The significance of this ratio was assessed using a sign test.

## Results and discussion

### ANNs accurately infer branch lengths generated by uniform distributions

We sought to compare the performance of several artificial neural network (ANN) estimators of branch lengths to that of maximum likelihood (ML) estimation (see Methods). First, as a simple scenario we evaluated each methods’ performance on the tasks of estimating the branch lengths ***b*** of a quartet tree sampled from generic uniform distributions with different minimum and maximum parameter values and MSAs simulated under JC model (see **Table 1**). In these experiments, the branch lengths ranged from very short (averaging 0.0005 substitutions per site) to very long branches (averaging 5.5 substitutions per site). Biologically, such contrasting scenarios reflect extremely slow versus fast uniformly evolving sequences or alternatively they can represent recent radiations versus deep taxonomic divergences. From a methodological perspective these scenarios encompass difficult parameter spaces, because MSAs simulated along very short trees will have limited phylogenetic information, whereas MSA’s simulated using long trees will be highly saturated with substitution events, which will erode phylogenetic signal in sequences that constitute the MSA, making them all appear highly dissimilar [30]. Thus, both of these scenarios may compromise a method’s ability to infer tree topology and ***b*** accurately [31,32]. For example, it is expected that multiple hits, if not accounted for, [12,33] or cases of “deep-time” substitutional saturation [34] will lead to underestimation of ***b***.

**Fig 2:**
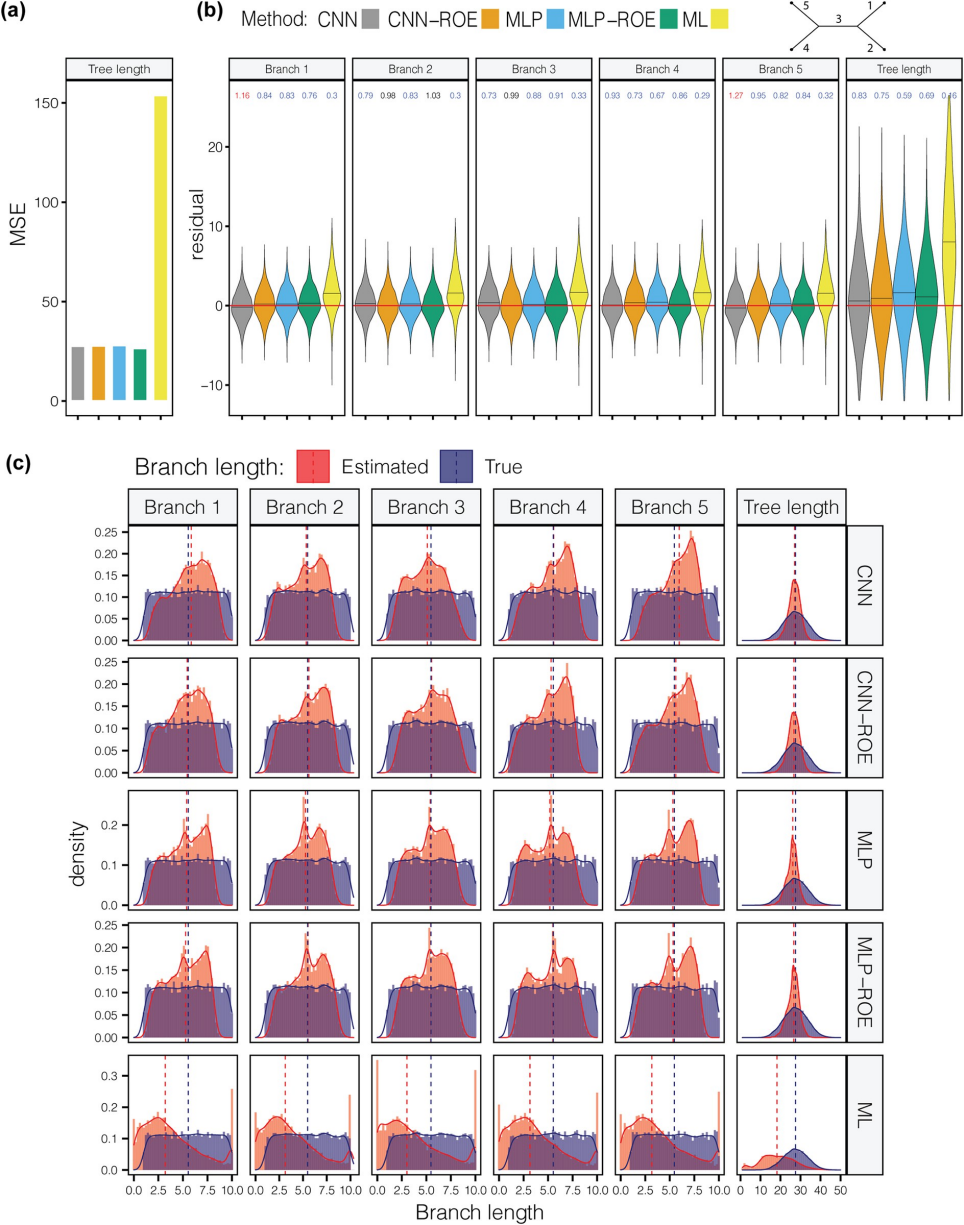
Performance of methods for long branch lengths generated using uniform distribution with minimum and maximum parameters of 1 and 10, respectively. (a) Comparison of mean squared error (MSE) for all methods. All artificial neural network models show superior performance relative to maximum likelihood. (b) The violin plot that shows the distribution of residuals (i.e. difference between true and inferred branch lengths) for each method. The value above each violin represents the ratio of overestimated and underestimated branches. The values of ~1 indicate an equal number of over- and underestimates, <1 indicate the underestimation is more common, and >1 indicates that overestimation is more common. The colored values represent statistically significant underestimation (blue) or overestimation (red). The horizontal red line marks 0. The black horizontal line within each violin shows the median. (c) Comparison of true (blue) and predicted (red) branch length distributions. Dashed lines mark the position of the median. CNN = convolutional neural network; CNN-ROE = convolutional neural network–regression of observed on estimated values; MLP = multilayer perceptron; MLP-ROE = multilayer perceptron–regression of observed on estimated values; ML = maximum likelihood.

For the *U*(0,0.001) experiment, ANNs outperformed ML across all branches and total tree lengths (S1 Table). Interestingly, the ANNs tended to infer branch lengths closer to the mean of *U*(0,0.001) which is equal to 0.0005 (S1A Fig). ML, on the other hand, showed exceptionally poor performance with highly inaccurate branch predictions (S1 Table). All methods underestimated branch lengths (S2A Fig). For the *U*(1,10) experiment we observed that all ANNs exhibit similar performance across branches and tree lengths (**Fig 2A and** S1 Table), whereas ML shows markedly inferior inferences (S1 Table). For example, for total tree length estimates the MSE, MAE, Spearman’s *ρ*, and *bias* for all ANN architectures (with the marginal superiority of MLP and MLP-ROE) were ~28, ~4.2, ~0.44 and ~3.6, respectively but ML’s measures were ~154, ~10, ~0.27 and 9.2, respectively. In this case, as a general trend we noticed that all methods underestimate ***b*** (**Fig 2B**), however for branches 2 and 3 CNN-ROE did not show any such systematic bias. A comparison of the true and estimated means of branch length distributions reveals that the average ***b*** and total tree length was inferred relatively accurately by ANNs (**Fig 2C**). ML, on the other hand, underestimates ***b***, so that the estimated density of ***b*** is mostly concentrated near the lower boundary of *U*(1,10) with some predictions being even lower (**Fig 2C**). In other experimental scenarios (S1 Table and Figshare project available at https://doi.org/10.6084/m9.figshare.21514272.v3), the ANNs perform well, and almost always outperformed ML or exhibited similar accuracy.

Together, the distribution of residuals and comparison between true and predicted ***b*** distributions can inform about the tendency and magnitude of a method’s over- or underestimation of ***b***. As a general trend, we noticed that ML produces an excess of severely overestimated ***b***, which can be seen in the left tail of predicted ***b*** distribution that goes beyond the boundaries (across all simulation scenarios that use uniform distribution) of the true ***b*** distribution (S1A–S1E Fig). Interestingly, ANNs tend to generate predicted ***b*** distributions within the theoretical distribution boundaries, which suggests that ANNs are not inclined to severely overestimate/underestimate ***b***. However, our examination of residual plots (S2A–S2E Fig) revealed that in most of the cases, all methods are prone to underestimating ***b*** although we stress that the magnitude of underestimation is substantially lower for the ANNs than ML. Additionally, ANNs are more efficient estimators of ***b***, since without any exception across all the experiments with uniform distributions ANNs have always smaller MSE values for all branch lengths and the total tree length (S1 Table).

### ANNs accurately infer branch lengths generated by exponential distributions

**Fig 3:**
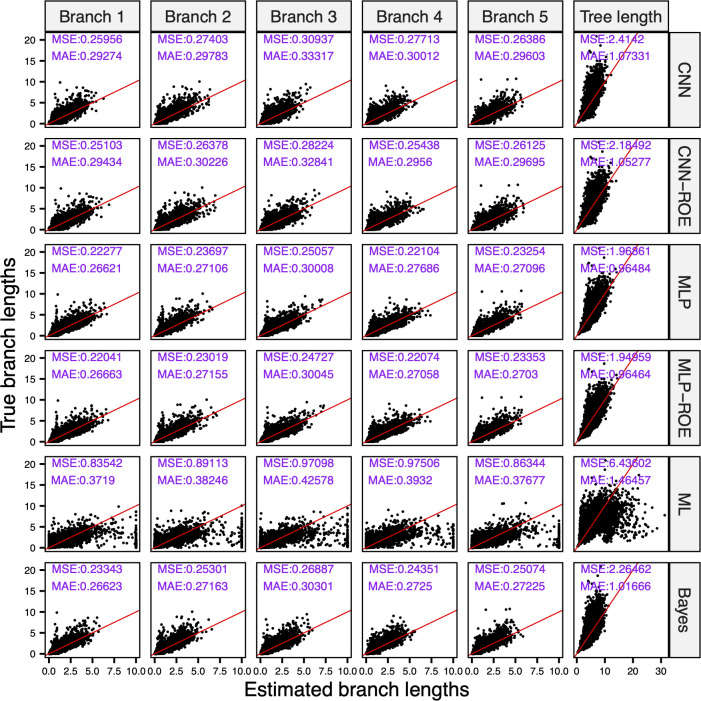
Correlation between estimated and true branch lengths drawn from exponential distribution with mean of 1. The majority of artificial neural network models show higher efficiency and accuracy than the maximum likelihood and Bayes methods. CNN = convolutional neural network; CNN-ROE = convolutional neural network–regression of observed on estimated values; MLP = multilayer perceptron; MLP-ROE = multilayer perceptron–regression of observed on estimated values; ML = maximum likelihood; The numbers in purple indicate mean squared error (MSE) and mean absolute error (MAE). Red line indicates perfect linear relationship.

We next evaluated each method’s performance on trees whose branch lengths were drawn from exponential distributions with means of 0.01, 0.1 and 1. The exponential distribution is commonly used as a prior for ***b*** in phylogenetic Bayesian frameworks [35] and is more biologically meaningful than a uniform distribution as it better fits the distribution of branch lengths in empirical datasets [15,36]. For each of these exponential branch length distributions, we generated two sets of MSAs: one using the JC substitution model and one using the GTR model (see **Table 1**). Generally, methods’ performances are similar between experiments that use JC and GTR models, but with slightly better outcomes achieved for the former case (S2 Table and Figshare project available at https://doi.org/10.6084/m9.figshare.21514272.v3). Here we describe the results obtained for the MSAs generated under JC. The performance across ANNs and ML was comparable for the experiment with exponential means of 0.01 and 0.1 (S2 Table). However, for the experiment with exponential mean of 1, ANNs significantly outperformed ML, showing error rates as measured by MSE that were an order of magnitude lower than that of ML (**Fig 3**). The disparity between MAEs was smaller, which is best explained by ML’s tendency to occasionally produce wildly inaccurate predictions, which have a larger impact on MSE than MAE. Interestingly, for all methods except the CNN without ROE (see Methods), the eCDF of predicted total tree lengths matched the true eCDF of total tree lengths (KS test, all *P* > 0.05); however, this result varied for individual branch lengths (S2 Table and Figshare project available at https://doi.org/10.6084/m9.figshare.21514272.v3).

Comparing the performance of ANNs to Bayesian estimation in the experiment with exponential means of 0.01 and 0.1 we found that the Bayesian approach exhibits similar or marginally better MSEs and MAEs, however it tends to be more biased than most of the ANNs (S2 Table). For example, the estimation bias for the total tree length was 0.0016, 0.0084 and 0.795 for MLP-ROE, whereas for Bayesian approach the bias was 0.0023, 0.0158 and 1.185 in the experiments with exponential means of 0.01, 0.1 and 1, respectively. In the experiment with exponential mean of 1, the MLP and MLP-ROE, outperformed Bayes in terms of MSE and MAE, i.e. MLP-ROE’s MAE of 0.965 and MSE of 1.95 vs. Bayes’ MAE of 1.017 and MSE of 2.26 (**Fig 3** and S2 Table).

### ANNs show elevated accuracy for trees with long branches

Trees with heterogeneous branch lengths can significantly deteriorate the performance of phylogenetic methods. Classical examples of such regions of branch length parameter space include the Felsenstein zone [16] and the Farris zone [17], where even statistically consistent methods may not exhibit good accuracy in tree topology estimation, depending on an MSA’s length. In fact, deep learning approaches may also suffer in such regions, especially in the Felsenstein zone [7]. However, this drawback can be ameliorated by increasing the alignment length, and in the case of deep learning methods, altering the composition of the training set to include more examples from these challenging regions of the parameter space. Inference of branch lengths can be also affected within these regions: it has been shown that branch length heterogeneity can decrease a method’s efficiency (i.e. its accuracy on smaller MSAs) and cause estimation artifacts such as non-independence of inferred ***b*** [6].

**Fig 4:**
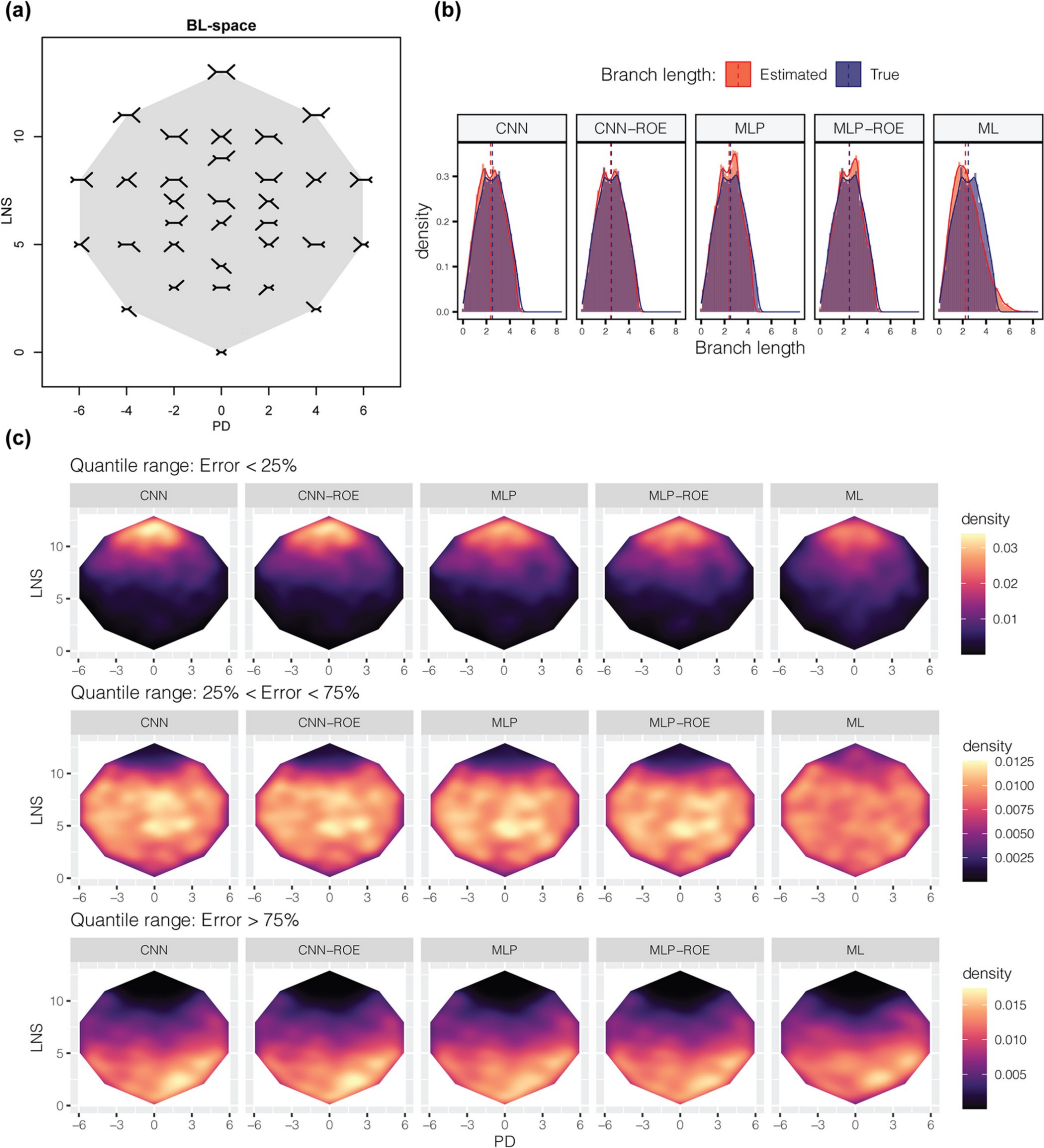
Performance within branch length heterogeneity space (BL-space). (**a**) The branch length space (BL-space). Each tree in the BL-space marks the location of the extreme cases where short branches in the image represent branch lengths equal to 0 and long branches represent lengths of 1. The shaded area shows the boundaries of the BL-space. PD = pairwise difference, LNS = tree length (L) + lengths of neighboring branches (NS). See Suvorov et al. (2020) for more detail [7]. (**b**) Comparison of true (blue) and predicted (red) total tree length distributions. Dashed lines mark the position of the median. (**c**) Distribution of average tree errors (absolute value of a difference between true and predicted branch lengths divided by the true branch length). Each panel corresponds to error quantile ranges. CNN = convolutional neural network; CNN-ROE = convolutional neural network–regression of observed on estimated values; MLP = multilayer perceptron; MLP-ROE = multilayer perceptron–regression of observed on estimated values; ML = maximum likelihood.

We previously described a distribution of branch lengths that captures a wide array of tree configurations, including those in the Farris and Felsenstein zones and other challenging regions of the parameter space. This distribution, which we call the BL-space (**Fig 4A**), is described in detail in Suvorov et al. (2020) and briefly in the Methods section [7]. Our simulations show that ANNs exhibit overall significantly better performance on MSAs with branch lengths drawn from this BL-space in both JC and GTR testing datasets in terms of the MSE, MAE, *ρ* and *bias* across all branches of our quartet topologies and the total tree lengths (S3 Table and Figshare project available at https://doi.org/10.6084/m9.figshare.21514272.v3). Although none of the examined methods were able to produce distribution of ***b*** estimates that match true distribution of ***b*** in BL-space (KS test, all *P* < 0.05), the *bias* and *D* statistic were noticeably smaller for ANNs than for ML (S3 Table). We also note that ROE consistently improves the accuracy of MLP and CNN as measured by MSE, MAE and *bias* (S3 Table). More detailed comparison of the true and estimated ***b*** distributions revealed that ANN models tend to infer ***b*** that more closely follow the shape of the true distribution of tree lengths (**Fig 4B**) as well as the shapes of ***b*** distributions in individual branches (S3 Fig), than do the estimates from ML. Moreover, ***b*** predicted by ANNs do not significantly extend beyond the domain of the BL-space, i.e. ***b***∈[0,1] and tree lengths ∈[0,5], whereas noticeable fraction of ML estimates goes beyond the upper bound of the BL-space domain. This observation suggests ***b*** overestimation by ML, but also that ANNs do not frequently produce estimates outside of their training range, a notion we examine further below. Then, we investigated the distribution of relative error (∑i|bi−bi^|bi, where *b*_*i*_ and bi^ are true and estimated values for branch *i* for each tree) to see if methods show some performance differences affected by branch length heterogeneity that is not governed merely by the size of the branch. Consistent with our previous results for the uniform distributions, ANNs tend to produce lower relative estimation errors than ML for trees with longer branches (**Fig 4C**). This observation is exemplified by the panels which show the density of trees that had the lowest relative estimation error (error < 25% quantile), which is highest towards the top of the BL-space for ANNs, where trees consisting entirely of long branches reside (**Fig 4C**). Although, we did not observe any dramatic performance biases, all estimation methods had larger errors (error > 75% quantile) for the trees with small ***b*** on average and within the Felsenstein zone (**Fig 4C**, bottom and lower middle parts, respectively, of the BL-space). These results are not surprising since trees with very short branches used in our simulations are expected to generate MSAs with very few variable sites, whereas Felsenstein zone is known to cause long branch attraction artifact for topology estimation problems and may also result in inaccurate ***b*** inference.

### ANNs accurately estimate branches that connect to a root node

We simulated branch lengths using birth-death models assuming a strict clock on a rooted quartet topology with a balanced tree shape i.e. ((A,B),(C,D)). Since this process generated rooted trees, the MSAs were generated using 3.3b and UNREST substitution models which are non-reversible and thus are able to account for the position of a root node that splits a branch into two branches with identifiable lengths [37].

Since the overall performance of ANNs and ML on MSAs generated under the 3.3b or UNREST models were similar (S4 Table and Figshare project available at https://doi.org/10.6084/m9.figshare.21514272.v3), here we report the results only for model 3.3b. Additionally, the relative extinction parameter *ϵ* did not have a large effect on these methods’ performance (S4 Table). ANNs exhibited superior estimation accuracy compared to ML. Specifically, ML struggled to infer the lengths of branches that connect to the tree root node under non-reversible substitution models. The predicted ***b*** that are connected to a root node by ML were biased and had notably poorer correlation (however significant, SR test, all *P* < 0.05) with true root node ***b***, as exemplified by coefficients *ρ*, than those estimated by ANNs (S4 Table). The ML estimates of terminal branches were notably better than internal ones across all parameter settings but still inferior to the ANNs’ estimates. In contrast, ANNs were able to accurately infer all ***b***, including the lengths of internal branches connected to the root, which in turn implies that ANNs are capable of inferring the position of the root of a tree. The correlations between predicted and true ***b*** values, as well as between predicted and true total tree lengths, were strong and significant (SR test, all *P* < 0.05) with *ρ* > 0.92 across all simulation scenarios. In most of the cases the MSE and MAE metrics for ANNs were an order of magnitude lower than those reported for ML (S4 Table). All methods of branch-length estimation exhibited better performance for trees with root node age of 50 Mya or 100 Mya (S4 Table). This result is expected, because for these trees the average length of the path from the terminal node to the root node will be 0.05 substitutions/site or 0.1 substitutions/site, respectively. Under such parameter settings, simulated MSAs will have a sufficient number of variable sites but will not be too diverged to reliably estimate ***b***, as opposed to the other cases we tested (root ages of 10 Mya and 200 Mya) which have average path lengths of 0.01 or 0.2, respectively, which may produce less informative MSAs. Also, we note ANNs correctly infer that sister terminal branches (i.e. A and B or C and D) should have identical lengths as a result of tree ultrametricity resulting from our simulations that use strict clock. Specifically, our analysis of ***b*** predicted for A and B (or C and D) in the scenario with *ϵ* = 0.5, *a* = 100 show that their lengths are highly correlated (SR test, ~1, *P* = 0).

The notion that ANNs are able to precisely infer the tree root is particularly encouraging, since the problem of finding an appropriate outgroup remains a challenging task in practice [38,39]. Moreover, ANNs recognize clock-like ***b*** directly from MSAs, a property that can be potentially used to identify clock models directly from MSAs without the need to reconstruct phylogenetic trees. In fact, recent study shows that a machine learning framework can effectively distinguish between independent and autocorrelated branch rate models using summary statistics extracted from the trees [40]. An advantage of using CNN architectures for this task is that they would not require any pre-defined summary statistics.

### Model misspecification and performance of ANNs outside of training parameter space

Here we describe evaluation of our methods under two conditions of model/parameter misspecification: (i) misspecification where training MSAs were generated under JC, whereas testing MSAs were generated under GTR (and vice versa) and (ii) testing ANNs on scenarios where the distribution of branch lengths used during training differs from that of the test set (**Table 1**).

Model selection plays an important part in phylogenetic inference. If a chosen model is a poor fit to molecular data, it may lead to erroneous inference of tree topology and/or branch lengths, (but see Spielman 2020) [41]. Although topology estimation is less sensitive to model misspecification if the most parameter-rich model is specified, branch-length estimation can be more sensitive [42]. In general, all methods’ performance was less impacted by model misspecification when trained on MSAs generated under GTR (or set to use the GTR model, in the case of ML) and testing MSAs were generated under JC than in the opposite scenario (S5 Table). This result is not surprising since the JC model is nested within the GTR model. Interestingly, we found that the CNN and CNN-ROE approaches were more susceptible to model misspecification than both ML, MLP and MLP-ROE methods, especially with ***b***~*Exp*(0.01) (S5 Table and Figshare project available at https://doi.org/10.6084/m9.figshare.21514272.v3). A similar trend was observed for ***b*** inferred by ML. In the opposite setting however, when methods were trained/set to be applied to data generated under JC model but were instead tested on MSAs generated under GTR, ML produced comparable results with the ANNs. ANNs also exhibited greater robustness to model misspecification and produced notably better estimates than those produced by ML for the other two branch length distributions, i.e. *Exp*(1) and *Exp*(0.1).

Next, we examined the impact of misspecification of the distribution of branch lengths used during training. The ANNs showed unsatisfactory behavior in experiments where we trained ANNs on MSAs simulated using trees with ***b*** sampled from *U*(0.1,1) and tested on *U*(1,2) and vice versa. In both cases, the ANNs failed to accurately predict branch lengths, with the distributions of estimated ***b*** falling entirely outside of the true ***b*** distribution with no overlap (S4 Fig and S6 Table).

However, these specific results do not necessarily show that ANNs poorly generalize target distribution. Instead, they only indicate that if the training ***b*** distribution does not encompass the true values, ANNs will not be capable to accurately predict ***b*** on the test set. One can make parallels with Bayesian inference where it is assumed that the domain of the appropriate prior distribution includes the true values of the parameter and thus have non-zero probability. In order to show that ANNs can generalize a target distribution well, we partitioned *Exp*(1) test distribution from the “exponential” experiment (see Table 1) into 20 quantiles and then calculated MSE and MAE metrics for each quantile partition for each method (S5 Fig). In general, ANNs exhibit accuracy that is higher than ML and comparable to Bayesian estimation, even in the upper tail of the exponential distribution where the density of branch lengths in the training data has larger variance. This suggests that ANNs require only a limited number of data points from low density regions of the training distribution to adequately generalize the true ***b*** distribution. This would imply that, so long as the true ***b*** lies within a region that is covered by the training distribution, even at a low density, misspecification of branch-length distributions will not have a dramatic effect.

Next, we assessed the performance of ANNs that were trained on the *Exp*(1) ***b*** distribution but tested under *Exp*(0.1). In this setting, the ANNs tended to be more biased by overestimating branch lengths and thus were less accurate. For example, for the best performing ANN model in the *Exp*(0.1) experiment (see Table 1), i.e. MLP-ROE, the bias increased from 0.008 to 0.335 and MSE for the total tree length increased from 0.0031 to 0.084 (S2 Table). In short, it appears that ANNs can be sensitive to the choice of the training distribution and hence, when using them one must take care to ensure that (i) the distribution of branch lengths used in training is sufficiently broad, so that it is likely to encompass the true ***b*** and/or (ii) train ANNs using a mixture distribution to better generalize their performance. We also note that the choice of ***b*** distribution prior can significantly affect phylogenetic inference in the Bayesian framework as well [43–45]. However, approximating the true branch-length distribution during training is not necessary for achieving superior performance to ML even in low density regions of this distribution.

### Effects of tree-shape balance on branch-length estimation

**Fig 5:**
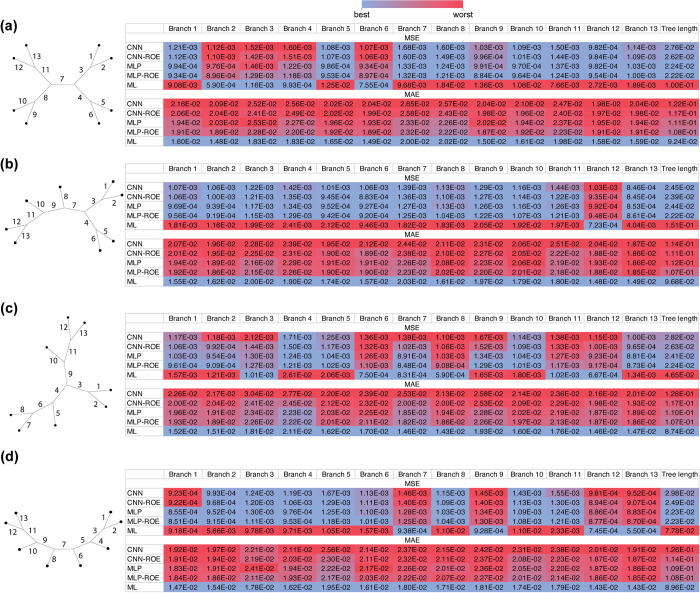
Effects of the shape balance on accuracy of estimated branch length using Mean Squared Error (MSE) and Mean absolute Error (MAE) metrics across methods. (**a**) The table represents a comparison of different methods’ performance for each branch (column names in the tables correspond to the numbered branches of a tree) and total tree length for balanced unrooted tree; panels (**b**) through (**d**) show the same for trees with increasing degrees of imbalance, with the most imbalanced tree, i.e. tree with pectinate (aka caterpillar) shape shown in (**d**). Color scheme ranks MSE or MAE metrics across all methods for a given branch (or total tree length), with the best values for a given branch shown in blue and the worst values shown in red. CNN: convolutional neural network; CNN-ROE: convolutional neural network–regression of observed on estimated values; MLP: multilayer perceptron; MLP-ROE: multilayer perceptron–regression of observed on estimated values; ML: maximum likelihood.

Besides branch-length heterogeneity, the degree of tree-shape balance, i.e. the branching pattern, may pose problems for inference of branch lengths, especially for those branches that are situated deeper in a tree [46]. To investigate this, we first evaluated the performance of each method across all 8-taxon tree shapes, branches as well as total tree lengths using MSE and MAE (**Fig 5**). In general, for all examined 8-taxon tree shapes (**Table 1**) ANNs produced at least 4-fold lower MSE values than ML. ML, on the other hand, had slightly lower MAE values (at most 1.32-fold lower) than the ANNs across all branches (**Fig 5** and S7 Table, and Figshare project available at https://doi.org/10.6084/m9.figshare.21514272.v3). This observation suggests that although ML typically infers branch lengths slightly more accurately than ANNs, ML occasionally grossly mis-infers a branch’s length, which results in large MSE values. We note, however, that for quartet trees with branches drawn from *Exp*(0.1) and corresponding MSAs generated under GTR model (i.e. the same simulation conditions that were used for 8-taxon trees), the CNN-ROE, MLP and MLP-ROE outperformed ML both in terms of MSE and MAE metrics. For instance, total tree length MSE and MAE from MLP-ROE are ~9.07 and ~1.16 times better, respectively, than those obtained by ML (S2 Table and Figshare project available at https://doi.org/10.6084/m9.figshare.21514272.v3). Thus, the marginally better values of MAE for ML are observed only for our 8-taxon trees and not the smaller 4-taxon trees. Also, while comparing performance of different ANNs on 8-taxon trees, we noticed that MLP-based architectures tend to be marginally more accurate than CNNs (**Fig 5**).

**Fig 6:**
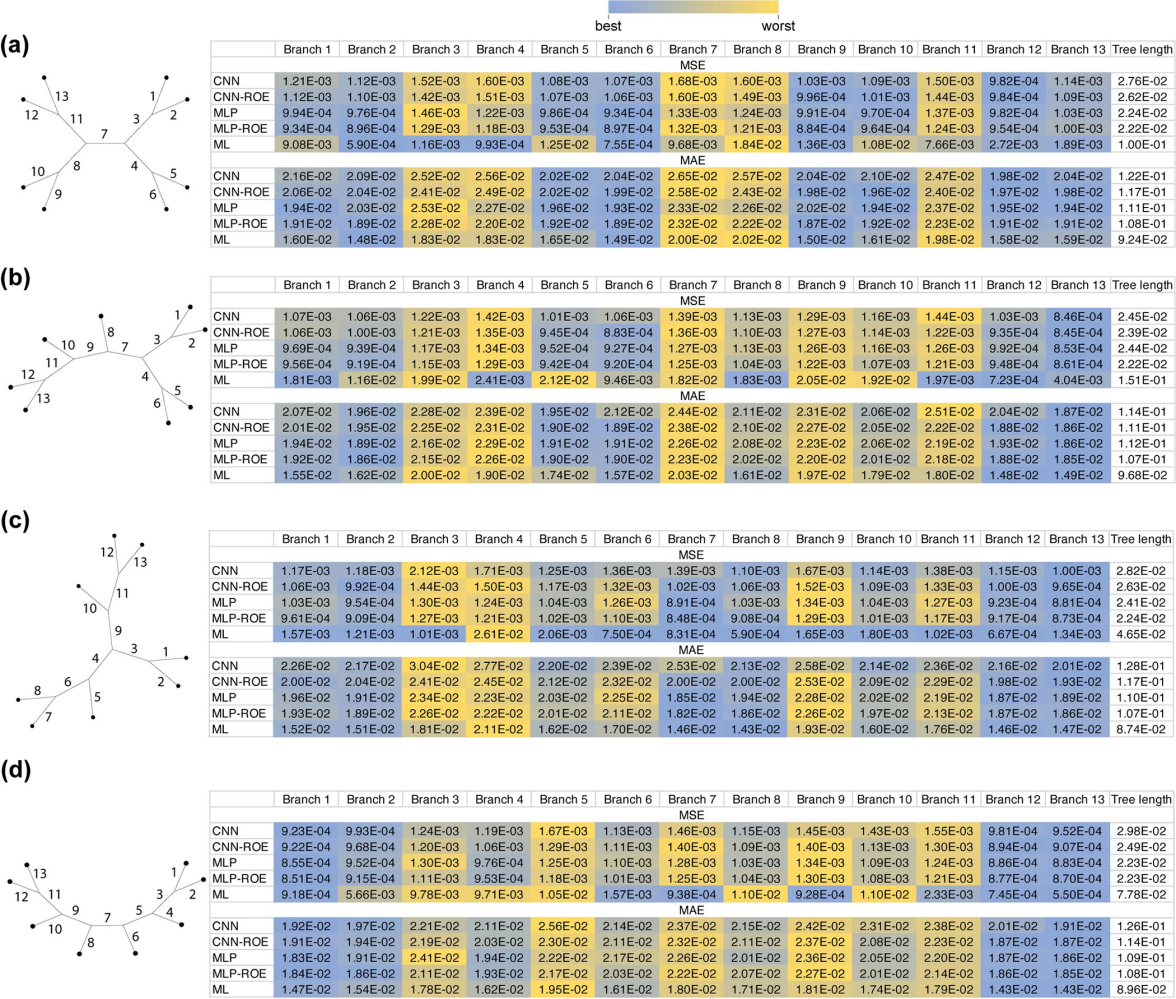
Effects of the shape balance on accuracy of estimated branch length using Mean Squared Error (MSE) and Mean absolute Error (MAE) highlighting differences between branches. Same as **Fig 5**, but with the color scheme showing which branches receive the lowest (blue) and highest (yellow) error metrics for a given method.

Next, for each method we examined the distribution of MSE and MAE values and their variances across all branches to see whether the ***b*** estimates show any variation in accuracy depending on the degree of tree balance and branch depths (**Fig 6** and S8 Table). As a general trend, we observed that MSE variance was nearly three orders of magnitude smaller for all ANNs than for ML, however the variance in MAE was similar or slightly smaller for ML when compared to ANNs, with the exception of MLP-ROE (S8 Table). Specifically, the MLP-ROE approach showed at least 1.2 times smaller MAE variance for all tree shapes compared to ML. Overall, we did not observe a systematic impact of tree shape on ***b***-estimation in terms of variance in MSE (or MAE): as we move from more symmetric to more asymmetric tree shapes in **Fig 6**, we do not observe an increase in error. Nevertheless, we observed a strong tendency for internal branches in a tree to have higher errors for ***b***-estimates in comparison with terminal branches for both the ANNs and ML. This pattern was especially clear in the distribution of MAE across tree branches (**Fig 6**). Taken together, our analyses suggest that 8-taxon trees represent a more difficult inferential problem than 4-taxon trees for ANNs, but on the other hand, ANNs on average showed adequate performance, and their superior MSE implies that they are highly reliable estimators in the sense that they do not produce the large errors sometimes observed for ML. We note here that it may be possible to further improve the ANN’s accuracy by constructing more optimal network architectures and/or using larger amounts of training examples simulated from the same parameter space [7].

### Concluding remarks and path forward

Here we investigated the possibility of deep learning models to estimate branch lengths on a fixed tree topology. Our ANNs showed excellent performance under a range of simulation conditions. Although ML is a highly accurate method for estimating branch lengths [12], in the majority of conditions considered here, including those regions of parameter space that may cause bias, ANNs provided superior branch length estimates than ML. Additionally, comparing the performance of our ANNs and ML with Bayesian estimation demonstrated that Bayes provides reliable branch length estimates comparable with those estimated by ANNs but with slightly higher bias. Interestingly, contrary to the findings in [12], ML produced inferior estimates to Bayes. This observation could be explained by the fact that Bayes performance is highly dependent on the choice of branch length prior distribution [45] and we used identical prior distributions to the ones used in simulation experiments, perhaps yielding somewhat overly optimistic estimates of the Bayesian method’s performance. The superior performance of ANNs to ML may be explained by the notion that ANNs itself can be viewed as Bayesian models where training datasets serve as a prior.

Due to the flexibility of artificial neural network architectures, such that they can be formulated to simultaneously execute classification and regression tasks, they could potentially be extended to co-estimate tree topology and branch lengths from MSAs. Since, ANNs are accurate estimators individually for both of these fundamental phylogenetic parameters (i.e. topology [7] and branch lengths as we showed in this study), it is possible that ANNs will exhibit similarly impressive accuracy when these parameters are estimated together, and future studies should explore this possibility. One current limitation of the ANN architectures examined here is that they cannot scale to larger numbers of taxa. However, explorations of alternative ANN architectures, or approaches that combined neural networks with aspects of more traditional tree optimization methods [11], may in due course overcome these limitations.

The encouraging performance of our implementations of ANNs suggest that these and similar methods can be extended to perform fossil calibrations, i.e., inference of branch lengths in absolute time. In principle, it is straightforward to incorporate fossil calibration data into the inputs for an ANN, along with multiple sequence alignments or summaries thereof, to estimate absolute divergence times on a fixed topology, provided that one can generate appropriate training data modeling the process that generates absolute branch length conditioned on the available calibration points. Also, it would be important to investigate the effects of indels on branch length estimation. Our previous study [7] showed that indel information can be effectively utilized toward more accurate tree topology inference within a machine learning framework. Thus, we expect that ANNs, which can implicitly construct indel models from training MSA by ANNs, may have the potential to improve branch length estimation relative to traditional methods that ignore indel information. Indel events provide useful information for evolutionary inference for highly diverged sequences [47,48] or in scenarios where the number of substitutions is insufficient for reliable estimation [49], so indels could have a positive effect on branch length estimation for trees with long or short branches.

Finally, we stress that generally ANNs provide more accurate estimates of long branch lengths than estimates from Bayes and ML (see the largest quantile of branch lengths in S5 Fig). Such long branches frequently occur in empirical datasets that contain deeply diverged taxa and/or fast evolving sequences. Due to the phenomenon of multiple substitutions occurring at the same site traditional methods may be prone to underestimate tree branch lengths from MSAs with highly saturated sequences, but it seems that ANN approaches that have been trained on data that include long branches tend to be more robust in such scenarios. Additionally, the ANNs’ accuracy for estimation of short branch lengths, which appear in datasets of closely related taxa and/or slowly evolving sequences, was comparable to that of the standard methods. We speculate that for MSAs with few substitutions, ANNs may require more training examples to learn a more accurate mapping from patterns in the alignment to the true branch lengths.

## Supporting information

S1 Fig Comparison of predicted and true branch length distributions generated by different uniform distributions. Branch lengths were generated by: (**a**) (0, 0.001); (**b**) *U* (0.001,0.01); (**c**) *U*(0.01, 0.1); (**d**) *U*(0.1, 1); (**e**) *U*(1, 10). Density plots represent true (blue) and predicted (red) branch length distributions. Dashed lines mark the position of the median. CNN = convolutional neural network; CNN-ROE = convolutional neural network–regression of observed on estimated values; MLP = multilayer perceptron; MLP-ROE = multilayer perceptron–regression of observed on estimated values; ML = maximum likelihood.
(PDF)

S2 Fig Comparison of residual distributions for branch length distributions generated by different uniform distributions. Branch lengths were generated by: (**a**) *U*(0,0.001); (**b**) *U*(0.001,0.01); (**c**) *U*(0.01,0.1); (**d**) *U*(0.1,1); (**e**) *U*(1,10). The violin plot that shows the distribution of residuals (i.e. difference between true and inferred branch lengths) for each method. The value above each violin represents the ratio of overestimated and underestimated branches. The values of ~1 indicate an equal number of over- and underestimates, <1 indicate the underestimation is more common, and >1 indicates that overestimation is more common. The colored values represent statistically significant underestimation (blue) or overestimation (red). The horizontal red line marks 0. The black horizontal line within each violin shows the median.
(PDF)

S3 Fig Comparison of predicted and true branch length distributions generated within branch length heterogeneity space (BL-space). The results were obtained under (**a**) JC and (**b**) GTR substitution models. Density plots represent true (blue) and predicted (red) branch length distributions. Dashed lines mark the position of the median. CNN = convolutional neural network; CNN-ROE = convolutional neural network–regression of observed on estimated values; MLP = multilayer perceptron; MLP-ROE = multilayer perceptron–regression of observed on estimated values; ML = maximum likelihood.
(PDF)

S4 Fig Misspecification of the distribution of branch lengths used during training. (**a**) ANNs were trained on MSAs simulated using trees with branch lengths sampled from (0.1,1) and tested on *U*(1,2). (**b**) ANNs were trained on MSAs simulated using trees with branch lengths sampled from (1, 2) and tested on (0.1, 1). Density plots represent true (blue) and predicted (red) branch length distributions. Dashed lines mark the position of the median. CNN = convolutional neural network; CNN-ROE = convolutional neural network–regression of observed on estimated values; MLP = multilayer perceptron; MLP-ROE = multilayer perceptron–regression of observed on estimated values.
(PDF)

S5 Fig Pooled mean squared errors (MSE) and mean absolute errors (MAE) across all tree branches for 20 quantiles of an exponential branch length distribution with the rate parameter of 1 (MSAs were generated under JC model). (**a**) An exponential distribution with rate parameter of 1 partitioned in 20 quantiles. (**b**) MSE values for each of the 20 quantiles. (**c**) MAE values for each of the 20 quantiles. Color scheme ranks MSE or MAE metrics across all methods for a given quantile. CNN = convolutional neural network; CNN-ROE = convolutional neural network–regression of observed on estimated values; MLP = multilayer perceptron; MLP- ROE = multilayer perceptron–regression of observed on estimated values; ML = maximum likelihood.
(PDF)

S1 Table Results from “Uniform” experiments (see Table 1). The name of each experiment follows this convention: *experiment_unif_{min}_{max}_{number of taxa}_{substitution model}*. The “*min*” and “*max*” correspond to the minimum and maximum parameter values of a uniform distribution. The following values were computed for each branch and tree length: mean squared error (MSE); mean absolute error (MAE); Spearman’s correlation coefficient (rho); Spearman’s correlation test *P* value (P_rho); bias (Bias); Kolmogorov-Smirnov test statistic *D* (D); Kolmogorov-Smirnov test *P* value (P_D). CNN = convolutional neural network; CNN-ROE = convolutional neural network–regression of observed on estimated values; MLP = multilayer perceptron; MLP-ROE = multilayer perceptron–regression of observed on estimated values; ML = maximum likelihood.
(XLSX)

S2 Table Results from “Exponential” experiments (see Table 1). The name of each experiment follows this convention: *experiment_exp_{rate}_{number of taxa}_{substitution model}*. The “*rate*” corresponds to the rate parameter of a uniform distribution. The following values were computed for each branch and tree length: mean squared error (MSE); mean absolute error (MAE); Spearman’s correlation coefficient (rho); Spearman’s correlation test *P* value (P_rho); bias (Bias); Kolmogorov-Smirnov test statistic *D* (D); Kolmogorov-Smirnov test *P* value (P_D). CNN = convolutional neural network; CNN-ROE = convolutional neural network–regression of observed on estimated values; MLP = multilayer perceptron; MLP-ROE = multilayer perceptron–regression of observed on estimated values; ML = maximum likelihood.
(XLSX)

S3 Table Results from “Branch-length heterogeneity space (BL-space)” experiments (see Table 1). The name of each experiment follows this convention: *experiment_mixb_{number of taxa}_{substitution model}*. The “*mixb*” denotes mixture beta distribution. The following values were computed for each branch and tree length: mean squared error (MSE); mean absolute error (MAE); Spearman’s correlation coefficient (rho); Spearman’s correlation test *P* value (P_rho); bias (Bias); Kolmogorov-Smirnov test statistic *D* (D); Kolmogorov-Smirnov test *P* value (P_D). CNN = convolutional neural network; CNN-ROE = convolutional neural network–regression of observed on estimated values; MLP = multilayer perceptron; MLP-ROE = multilayer perceptron–regression of observed on estimated values; ML = maximum likelihood.
(XLSX)

S4 Table Results from “Birth-death” experiments (see Table 1). The name of each experiment follows this convention: *experiment_bd_{relative extinction}_{age of the root node}_{clock rate}_{substitution model}*. The “*bd*” denotes birth-death model. The following values were computed for each branch and tree length: mean squared error (MSE); mean absolute error (MAE); Spearman’s correlation coefficient (rho); Spearman’s correlation test *P* value (P_rho); bias (Bias); Kolmogorov-Smirnov test statistic *D* (D); Kolmogorov-Smirnov test *P* value (P_D). CNN = convolutional neural network; CNN-ROE = convolutional neural network–regression of observed on estimated values; MLP = multilayer perceptron; MLP-ROE = multilayer perceptron–regression of observed on estimated values; ML = maximum likelihood.
(XLSX)

S5 Table Results from “Model misspecification” experiments (see Table 1). The name of each experiment follows this convention: *experiment_exp_{rate}_{number of taxa}_{substitution model of testing dataset/substitution model of training dataset}*. The “*rate*” corresponds to the rate parameter of a uniform distribution. The following values were computed for each branch and tree length: mean squared error (MSE); mean absolute error (MAE); Spearman’s correlation coefficient (rho); Spearman’s correlation test *P* value (P_rho); bias (Bias); Kolmogorov-Smirnov test statistic *D* (D); Kolmogorov-Smirnov test *P* value (P_D). CNN = convolutional neural network; CNN- ROE = convolutional neural network–regression of observed on estimated values; MLP = multilayer perceptron; MLP-ROE = multilayer perceptron–regression of observed on estimated values; ML = maximum likelihood.
(XLSX)

S6 Table Results from “Misspecified branch length distribution” experiments (see Table 1). The name of each experiment follows this convention: *experiment_unif_{number of taxa}_{substitution model}_train_{min}_{max}_test_{min}_{max}*. The “*min*” and “*max*” correspond to the minimum and maximum parameter values of uniform distributions that were used to simulate training and testing datasets. The following values were computed for each branch and tree length: mean squared error (MSE); mean absolute error (MAE); Spearman’s correlation coefficient (rho); Spearman’s correlation test *P* value (P_rho); bias (Bias); Kolmogorov-Smirnov test statistic *D* (D); Kolmogorov-Smirnov test *P* value (P_D). CNN = convolutional neural network; CNN-ROE = convolutional neural network–regression of observed on estimated values; MLP = multilayer perceptron; MLP-ROE = multilayer perceptron–regression of observed on estimated values; ML = maximum likelihood.
(XLSX)

S7 Table Results from “Exponential” experiments (see Table 1). The name of each experiment follows this convention: *experiment_exp_{rate}_{number of taxa}_{substitution model}_{balance} {tree topology in newick}*. The “*rate*” corresponds to the rate parameter of a uniform distribution. The value of “*balance*” indicates tree topology imbalance, where a bigger number corresponds to larger imbalance. The following values were computed for each branch and tree length: mean squared error (MSE); mean absolute error (MAE); Spearman’s correlation coefficient (rho); Spearman’s correlation test *P* value (P_rho); bias (Bias); Kolmogorov-Smirnov test statistic *D* (D); Kolmogorov-Smirnov test *P* value (P_D). CNN = convolutional neural network; CNN-ROE = convolutional neural network–regression of observed on estimated values; MLP = multilayer perceptron; MLP-ROE = multilayer perceptron–regression of observed on estimated values; ML = maximum likelihood.
(XLSX)

S8 Table Variance of mean squared errors (MSE) and mean absolute errors (MAE) across all branches in 8-taxon trees. The value of balance indicates tree topology imbalance, where a bigger number corresponds to larger tree imbalance. CNN = convolutional neural network; CNN- ROE = convolutional neural network–regression of observed on estimated values; MLP = multilayer perceptron; MLP-ROE = multilayer perceptron–regression of observed on estimated values; ML = maximum likelihood.
(XLSX)
