# Supplementary material for: Reliable estimation of tree branch lengths using deep neural networks
Source: PLoS Comput Biol. 2024 Aug 5;20(8):e1012337. doi: 10.1371/journal.pcbi.1012337 (PMC11326709; doi:10.1371/journal.pcbi.1012337)
Supplement: S1 Fig — Branch lengths were generated by: (a) (0, 0.001); (b) U (0.001,0.01); (c) U(0.01, 0.1); (d) U(0.1, 1); (e) U(1, 10). Density plots represent true (blue) and predicted (red) branch length distributions. Dashed lines mark the position of the median. CNN = convolutional neural network; CNN-ROE = convolutional neural network–regression of observed on estimated values; MLP = multilayer perceptron; MLP-ROE = multilayer perceptron–regression of observed on estimated values; ML = maximum likelihood. (PDF) [file pcbi.1012337.s001.pdf]

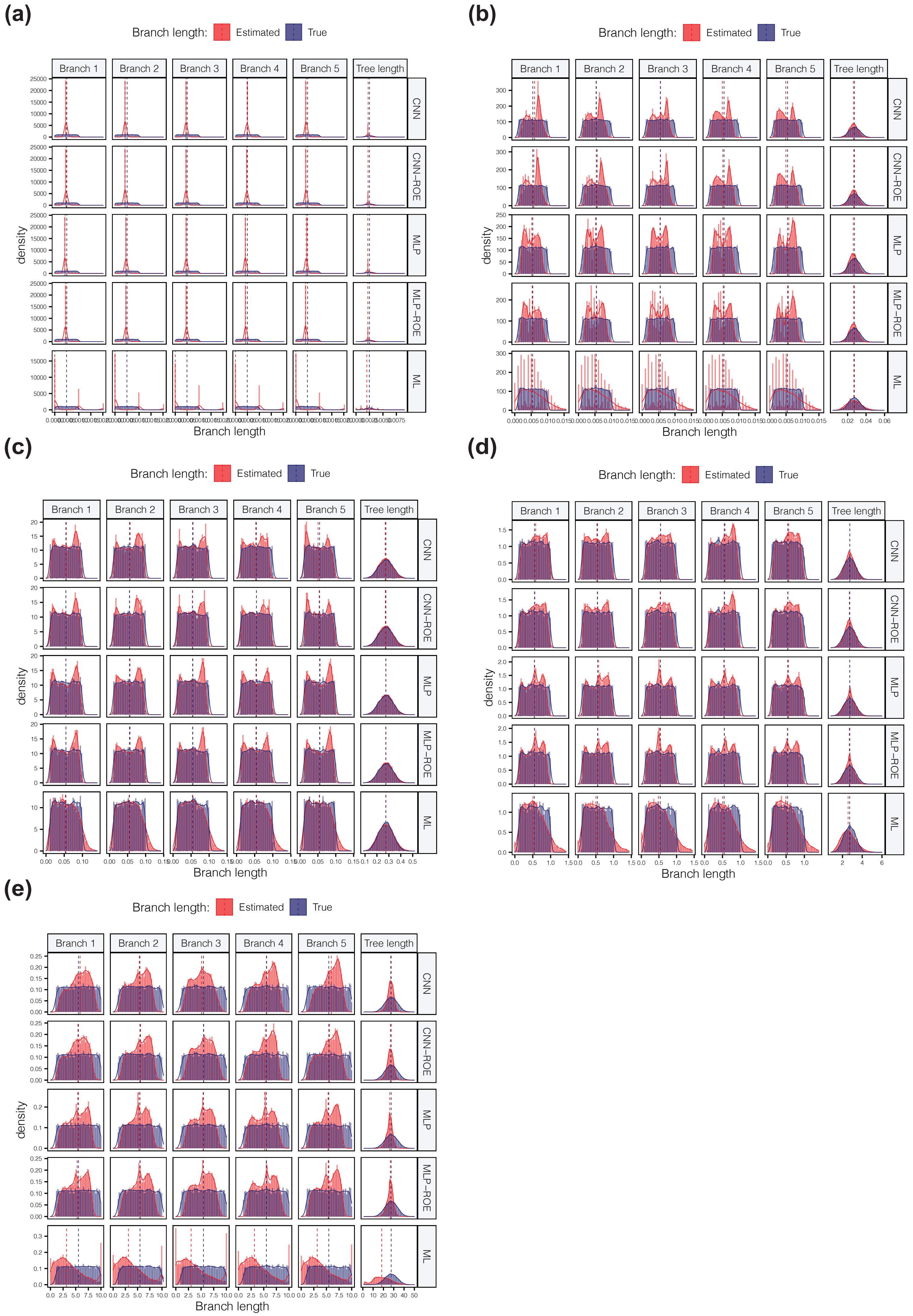

(a)
Branch length: Estimated True
Branch 1
Branch 2
Branch 3
Branch 4
Branch 5
Tree length
CNN
CNN–ROE
MLP
MLP–ROE
ML
density
Branch length
(b)
Branch length: Estimated True
Branch 1
Branch 2
Branch 3
Branch 4
Branch 5
Tree length
CNN
CNN–ROE
MLP
MLP–ROE
ML
density
Branch length
(c)
Branch length: Estimated True
Branch 1
Branch 2
Branch 3
Branch 4
Branch 5
Tree length
CNN
CNN–ROE
MLP
MLP–ROE
ML
density
Branch length
(d)
Branch length: Estimated True
Branch 1
Branch 2
Branch 3
Branch 4
Branch 5
Tree length
CNN
CNN–ROE
MLP
MLP–ROE
ML
density
Branch length
(e)
Branch length: Estimated True
Branch 1
Branch 2
Branch 3
Branch 4
Branch 5
Tree length
CNN
CNN–ROE
MLP
MLP–ROE
ML
density
Branch length
