# Supplementary figures and images for: Reliable estimation of tree branch lengths using deep neural networks
Source: PLoS Comput Biol. 2024 Aug 5;20(8):e1012337. doi: 10.1371/journal.pcbi.1012337 (PMC11326709; doi:10.1371/journal.pcbi.1012337)

**(a)**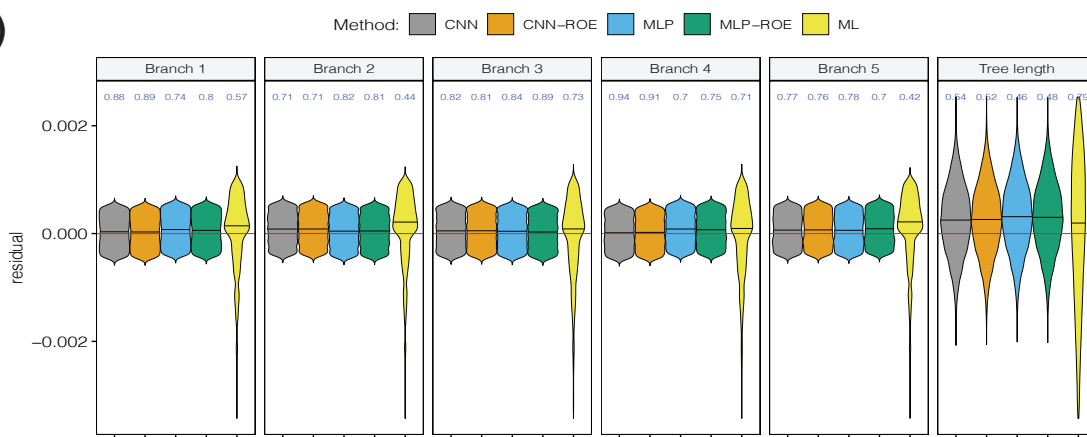**(b)**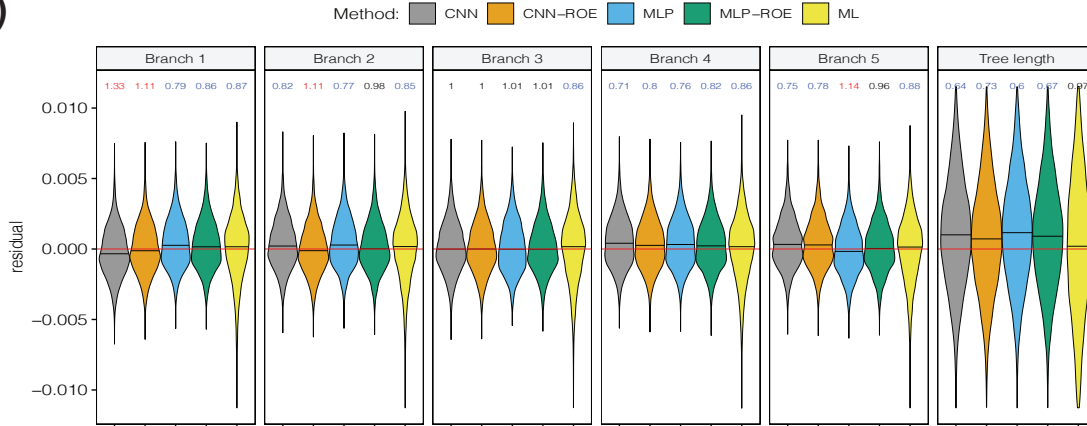**(c)**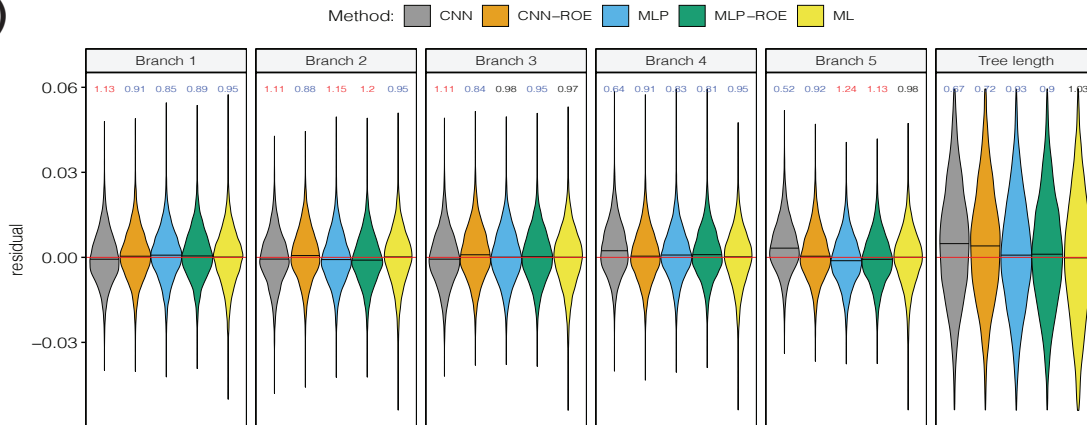**(d)**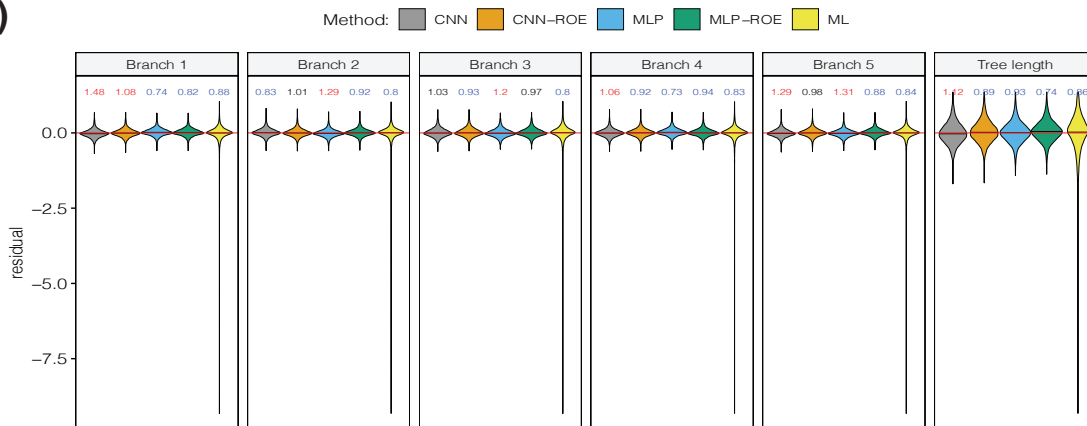**(e)**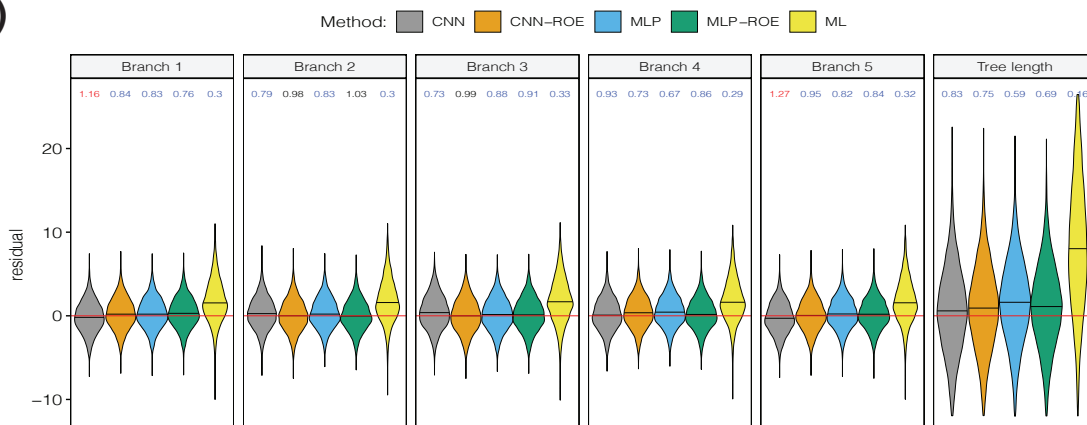

Supplement: S2 Fig — Branch lengths were generated by: (a) U(0,0.001); (b) U(0.001,0.01); (c) U(0.01,0.1); (d) U(0.1,1); (e) U(1,10). The violin plot that shows the distribution of residuals (i.e. difference between true and inferred branch lengths) for each method. The value above each violin represents the ratio of overestimated and underestimated branches. The values of ~1 indicate an equal number of over- and underestimates, <1 indicate the underestimation is more common, and >1 indicates that overestimation is more common. The colored values represent statistically significant underestimation (blue) or overestimation (red). The horizontal red line marks 0. The black horizontal line within each violin shows the median. (PDF) [file pcbi.1012337.s002.pdf]

**(a)**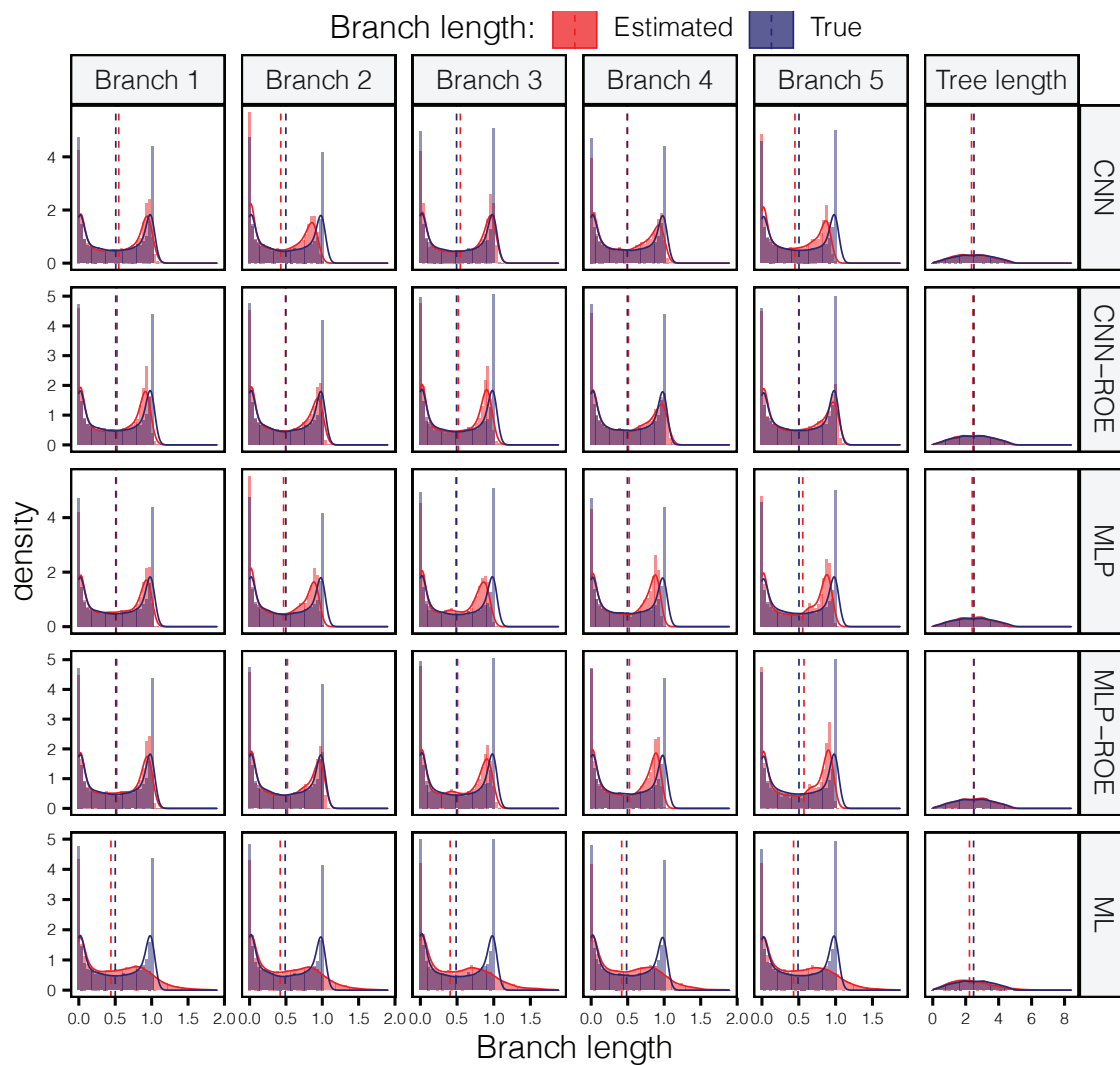**(b)**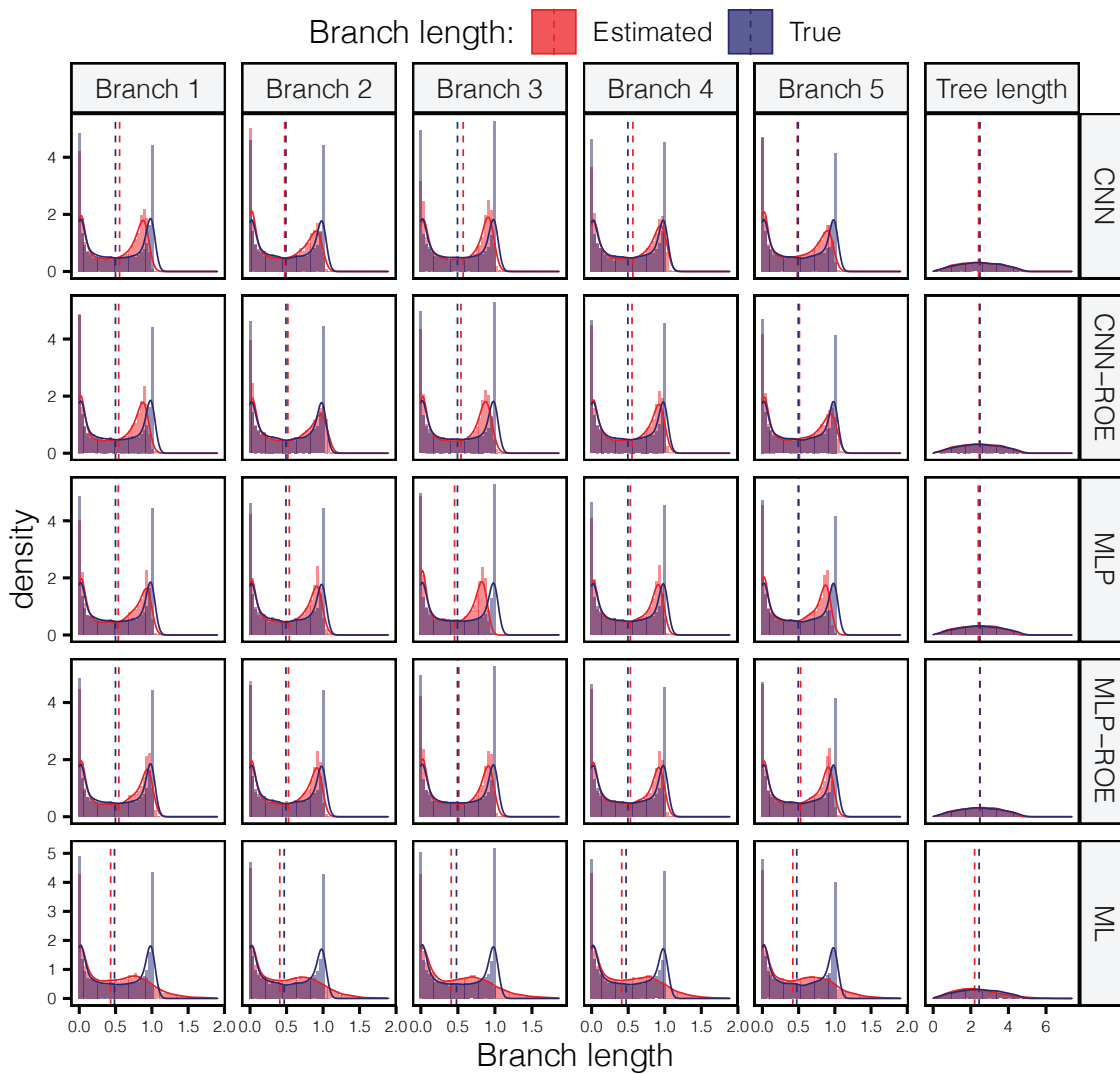

Supplement: S3 Fig — The results were obtained under (a) JC and (b) GTR substitution models. Density plots represent true (blue) and predicted (red) branch length distributions. Dashed lines mark the position of the median. CNN = convolutional neural network; CNN-ROE = convolutional neural network–regression of observed on estimated values; MLP = multilayer perceptron; MLP-ROE = multilayer perceptron–regression of observed on estimated values; ML = maximum likelihood. (PDF) [file pcbi.1012337.s003.pdf]

**(a)**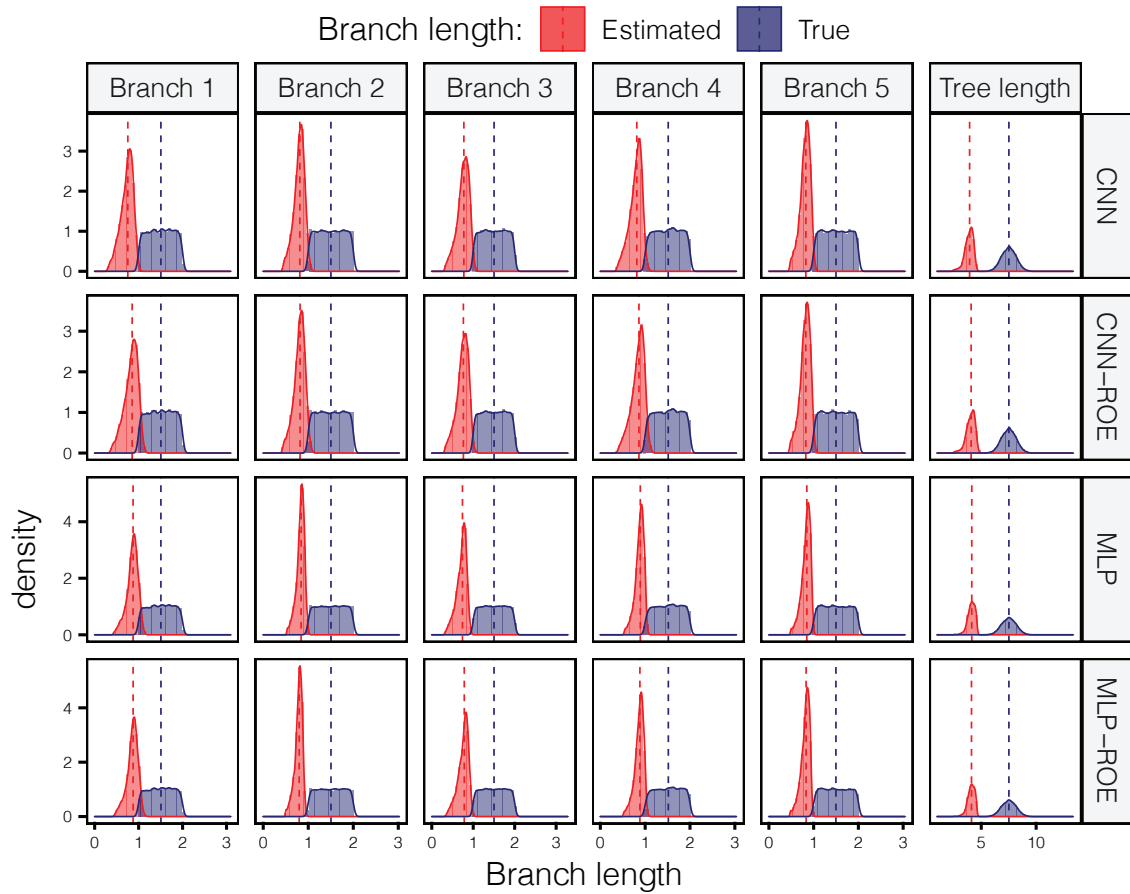**(b)**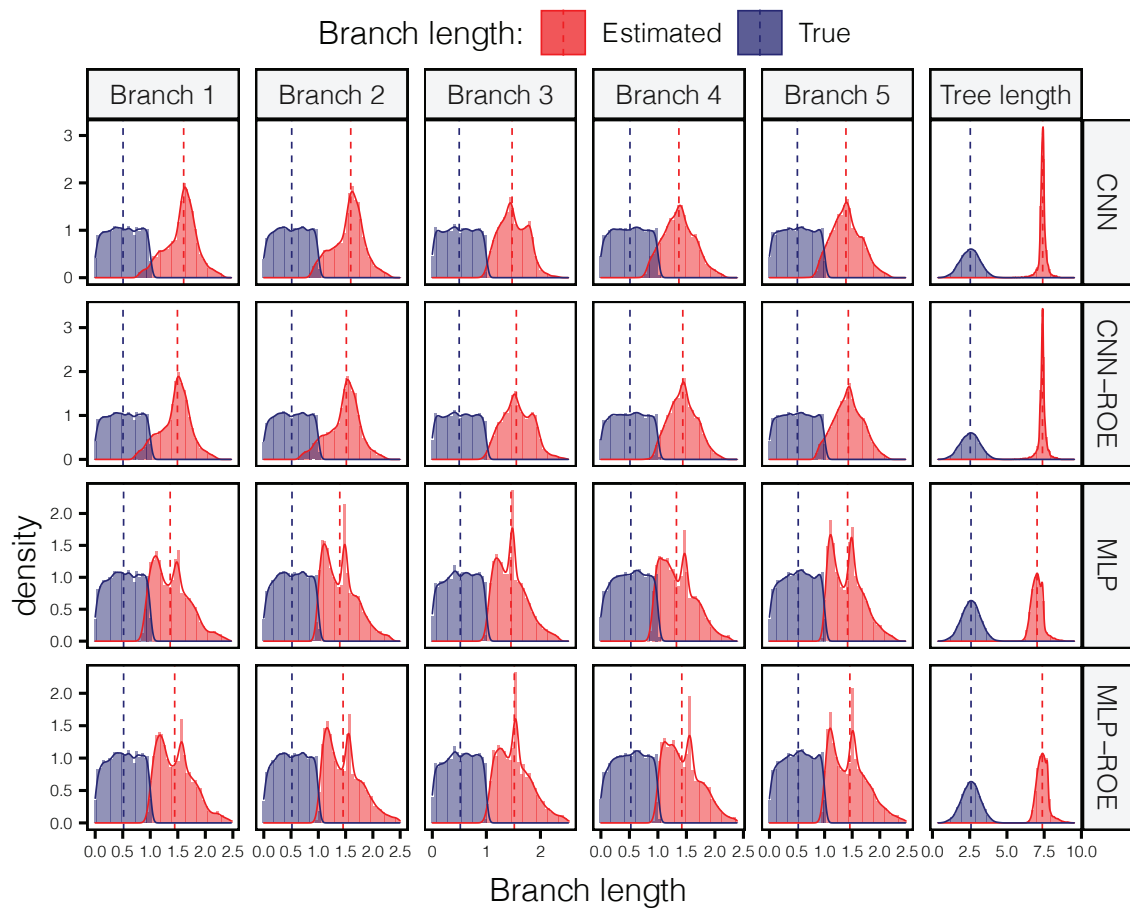

Supplement: S4 Fig — (a) ANNs were trained on MSAs simulated using trees with branch lengths sampled from (0.1,1) and tested on U(1,2). (b) ANNs were trained on MSAs simulated using trees with branch lengths sampled from (1, 2) and tested on (0.1, 1). Density plots represent true (blue) and predicted (red) branch length distributions. Dashed lines mark the position of the median. CNN = convolutional neural network; CNN-ROE = convolutional neural network–regression of observed on estimated values; MLP = multilayer perceptron; MLP-ROE = multilayer perceptron–regression of observed on estimated values. (PDF) [file pcbi.1012337.s004.pdf]
