# Supplementary material for: Reliable estimation of tree branch lengths using deep neural networks
Source: PLoS Comput Biol. 2024 Aug 5;20(8):e1012337. doi: 10.1371/journal.pcbi.1012337 (PMC11326709; doi:10.1371/journal.pcbi.1012337)
Supplement: S5 Fig — (a) An exponential distribution with rate parameter of 1 partitioned in 20 quantiles. (b) MSE values for each of the 20 quantiles. (c) MAE values for each of the 20 quantiles. Color scheme ranks MSE or MAE metrics across all methods for a given quantile. CNN = convolutional neural network; CNN-ROE = convolutional neural network–regression of observed on estimated values; MLP = multilayer perceptron; MLP- ROE = multilayer perceptron–regression of observed on estimated values; ML = maximum likelihood. (PDF) [file pcbi.1012337.s005.pdf]

(a)

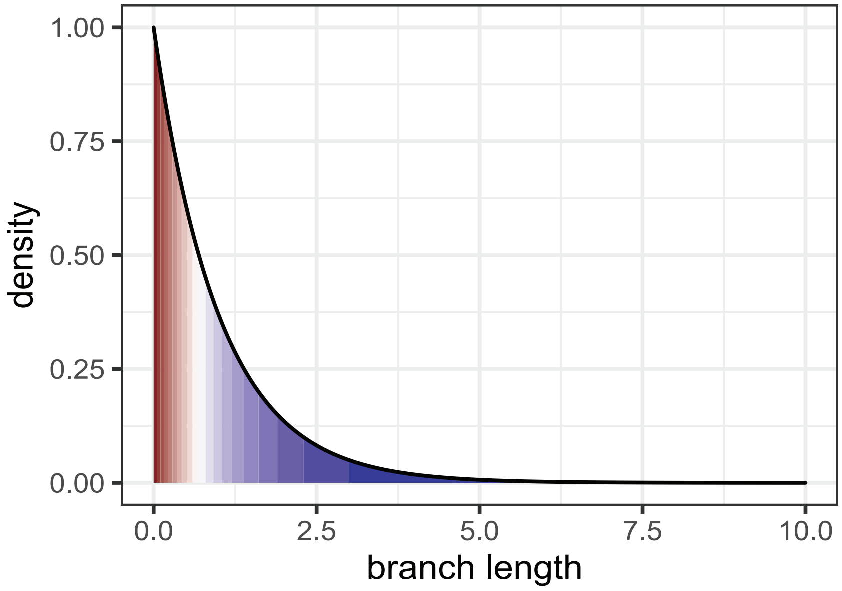

(b)

MSE						
quantile	CNN	CNN-ROE	MLP	MLP-ROE	ML	Bayes
1	4.81E-02	5.41E-02	4.00E-02	3.98E-02	1.75E-01	3.37E-02
2	5.03E-02	5.83E-02	4.14E-02	4.08E-02	5.07E-01	3.35E-02
3	4.57E-02	5.35E-02	3.34E-02	3.34E-02	2.76E-01	2.83E-02
4	4.90E-02	5.87E-02	3.70E-02	3.68E-02	2.71E-01	3.05E-02
5	4.64E-02	5.60E-02	3.54E-02	3.55E-02	4.10E-01	2.88E-02
6	5.57E-02	6.66E-02	4.02E-02	4.03E-02	3.58E-01	3.15E-02
7	5.58E-02	6.83E-02	4.39E-02	4.47E-02	4.72E-01	3.53E-02
8	5.40E-02	6.83E-02	4.39E-02	4.42E-02	6.54E-01	3.63E-02
9	6.73E-02	8.32E-02	5.26E-02	5.31E-02	8.33E-01	4.53E-02
10	6.94E-02	8.50E-02	5.66E-02	5.75E-02	7.58E-01	4.99E-02
11	8.54E-02	1.05E-01	6.44E-02	6.59E-02	7.44E-01	5.57E-02
12	9.07E-02	1.10E-01	7.96E-02	8.15E-02	5.89E-01	7.09E-02
13	1.03E-01	1.22E-01	8.98E-02	9.23E-02	1.01E+00	8.50E-02
14	1.33E-01	1.54E-01	1.16E-01	1.19E-01	8.37E-01	1.11E-01
15	1.57E-01	1.77E-01	1.46E-01	1.51E-01	9.57E-01	1.43E-01
16	2.15E-01	2.36E-01	1.90E-01	1.96E-01	1.06E+00	1.90E-01
17	2.85E-01	2.98E-01	2.64E-01	2.69E-01	1.17E+00	2.66E-01
18	4.17E-01	4.11E-01	3.78E-01	3.84E-01	1.26E+00	3.89E-01
19	7.24E-01	6.58E-01	6.22E-01	6.18E-01	1.89E+00	6.67E-01
20	2.83E+00	2.36E+00	2.31E+00	2.24E+00	3.97E+00	2.71E+00

best
worst

(c)

MAE						
quantile	CNN	CNN-ROE	MLP	MLP-ROE	ML	Bayes
1	1.25E-01	1.28E-01	1.19E-01	1.14E-01	8.99E-02	1.05E-01
2	1.18E-01	1.25E-01	1.11E-01	1.07E-01	1.36E-01	9.72E-02
3	1.14E-01	1.21E-01	1.03E-01	1.01E-01	1.21E-01	9.51E-02
4	1.25E-01	1.35E-01	1.15E-01	1.14E-01	1.29E-01	1.04E-01
5	1.34E-01	1.44E-01	1.22E-01	1.22E-01	1.55E-01	1.11E-01
6	1.48E-01	1.61E-01	1.37E-01	1.36E-01	1.63E-01	1.21E-01
7	1.56E-01	1.71E-01	1.48E-01	1.47E-01	1.85E-01	1.31E-01
8	1.65E-01	1.82E-01	1.55E-01	1.54E-01	2.24E-01	1.37E-01
9	1.84E-01	2.02E-01	1.72E-01	1.71E-01	2.66E-01	1.55E-01
10	1.95E-01	2.10E-01	1.80E-01	1.79E-01	2.76E-01	1.67E-01
11	2.18E-01	2.37E-01	1.93E-01	1.96E-01	3.02E-01	1.78E-01
12	2.30E-01	2.50E-01	2.14E-01	2.16E-01	3.11E-01	2.01E-01
13	2.45E-01	2.62E-01	2.29E-01	2.31E-01	3.86E-01	2.22E-01
14	2.87E-01	3.03E-01	2.65E-01	2.66E-01	4.07E-01	2.61E-01
15	3.11E-01	3.20E-01	2.99E-01	3.02E-01	4.59E-01	2.96E-01
16	3.69E-01	3.76E-01	3.46E-01	3.50E-01	5.22E-01	3.44E-01
17	4.30E-01	4.32E-01	4.09E-01	4.12E-01	6.08E-01	4.09E-01
18	5.22E-01	5.10E-01	4.89E-01	4.92E-01	7.02E-01	5.00E-01
19	6.90E-01	6.45E-01	6.14E-01	6.12E-01	9.07E-01	6.46E-01
20	1.34E+00	1.17E+00	1.14E+00	1.11E+00	1.48E+00	1.28E+00

best
worst
